# Knowledge-based intervention improves older adult recognition memory for novel activity, but not event segmentation or temporal order memory

**DOI:** 10.1038/s41598-023-45577-3

**Published:** 2023-10-31

**Authors:** Karissa B. Payne, Cristiane K. Brazil, Maria Apel, Heather Bailey

**Affiliations:** Kansas State University, 1114 Midcampus Drive, Manhattan, KS 66502 USA

**Keywords:** Psychology, Human behaviour

## Abstract

Although episodic memory declines with age, older adults are often able to make use of relevant knowledge to support episodic memory. More specifically, prior knowledge may support the perception of meaningful events through the process of event segmentation. We sought to test whether increasing older adults’ knowledge for novel activities (i.e., Tai chi, making gyozas) would improve segmentation and memory. We conducted an online, pre-registered intervention in which eighty older adults were recruited based on being novices in each of the targeted activities. Participants completed segmentation and memory tests before and after being randomly assigned to one of two interactive virtual workshops (learning how to practice Tai chi or make gyozas). Each workshop consisted of two one-hour sessions during which an expert provided information about the activity and demonstrated it in a step-by-step fashion. We found that the intervention led to increased learning and recognition memory for the trained activity; however, there were no significant improvements in segmentation behavior, free recall, or memory of sequential information. These findings indicate that either more knowledge training is necessary to affect segmentation, or that segmentation is guided by perceptual features in the environment rather than one’s conceptual understanding of the activity.

## Introduction

As we age, subtle shifts in our memory capabilities can significantly impact daily life, from recalling names to remembering recent events^1,2^. Although part of normal aging processes, research has been focused on better understanding the nuances of these changes and developing strategies to mitigate their effects and enhance the quality of life for older adults. To create interventions that combat these deficits, it is important to distinguish between different types of memory and how they vary with age. Specifically, older adults frequently maintain the ability to retrieve knowledge-based memory, or *semantic memory*^3^. In fact, some experiments show older adults are better able to use semantic memory compared to younger adults due to the accumulation of knowledge throughout an individual’s life^4,5^. In contrast, *episodic memory*, which is the ability to recall and recognize specific experiences—like someone’s name or where you placed your keys––tends to decline with age^6^. Some work has found evidence that retrieval mechanisms underlie differences in episodic memory^7,8^, whereas other work has found evidence for encoding deficits^9–11^. In this study we aimed to improve episodic memory in older adults through an intervention targeting an encoding process, specifically by targeting older adults’ ability to parse activity into meaningful event segments.

Because of the interdependence of episodic and semantic memory^12–15^, it is possible that the strength of older adults’ semantic memory can be leveraged to improve their episodic memory encoding. A wealth of research has shown that prior knowledge (i.e., semantic memory) supports episodic memory^16–24^, particularly when it can be used during encoding^25^. Additionally, there is evidence that older adults’ memory can benefit more from prior knowledge than that of younger adults^26–28^. An intervention that increases semantic knowledge for an activity could theoretically improve the encoding of contextually-similar events, and such an intervention should be especially impactful for older adults. Through this study, we will specifically examine *event segmentation* as an encoding strategy that could benefit from a knowledge-based intervention.

### Encoding episodic memory through event segmentation

Event Segmentation Theory (EST) posits that we mentally represent what is currently happening in a working memory model called an *event model*^29^. This event model is influenced by the sensory input we receive from the environment (sights, sounds, etc.) as well as relevant episodic memories and semantic knowledge. The event model helps to make predictions of what will happen in the near future^30^. When activities change and predictions are no longer accurate, EST claims that an event boundary––the end of one event and the beginning of another––is perceived and the activity is segmented into different events. This segmentation process can influence what content is encoded and what can later be retrieved.

Research has found that people tend to segment activities in similar ways, which has been observed in both in-person and online experiments^31^. However, there are individual and age-related differences in people’s event segmentation behavior. Specifically, prior work has shown that young adults segment more normatively (more similarly to one another) than older adults^32–36^ (though some studies have found conflicting results^30,37^), and that cognitively healthy older adults segment more normatively than older adults with early stage Alzheimer’s Disease (AD)^35,38^. Importantly, these age-related and AD-related differences in event segmentation may be associated with event memory. Prior research has largely found that people who are better able to detect event boundaries within an activity are also better able to remember the activity^34,35,38,39^, even after controlling for other cognitive factors such as processing speed and working memory^34^. Although this segmentation-memory association is often not observed in tasks measuring recognition memory^35,40^, it has more commonly been observed in recall memory tasks^34,39,41^. If the encoding process of event segmentation and event memory are related, it is possible that a knowledge-based intervention could improve both older adults’ event segmentation and event memory.

### Influence of top-down and bottom-up processing on segmentation

It is important to consider what influences the perception of event boundaries. Prior work has evaluated the effect of knowledge on event encoding and the results are mixed. A few studies using an expert-novice paradigm have found differences in segmentation ability within the experts’ domain of knowledge, such that experts segment at a different frequency^42,43^, and identify more similar coarse-grained events compared to novices^43,44^. Differences in segmentation between experts and novices suggest that prior knowledge provides a framework that helps people understand and construct episodic memory representations for ongoing events within their domain of expertise^45^. Conversely, other studies using top-down manipulations such as having people watch a film multiple times^46^, having people watch a film forward vs. backward^47^, and having fans from rival teams watch a soccer game^48^ have found little to no effect on event segmentation. These results may suggest that viewers’ event segmentation is heavily driven by bottom-up perceptual cues in the environment, such as motion^49^, individuals’ body positions^50^, and movements^51^. If event segmentation is primarily driven by perceptual information, then increasing knowledge will not influence segmentation ability.

However, it is important to consider that these prior studies have all included young adult samples. It is possible that older adults, compared to younger adults, may rely less on perceptual input from the environment and more upon their semantic knowledge and relevant experiences, due to the normal decline in perceptual processes and executive abilities with age^3,52,53^ In fact, this is what recent work with older adults from our lab has shown. In a series of experiments, we have evaluated young and older adults’ event segmentation and memory performance when they watched activities of familiar and unfamiliar activities. When older adults lacked prior knowledge, we observed the standard age-related deficit in segmentation and memory. Importantly, however, when older adults were able to access relevant knowledge (i.e., while viewing familiar activities) they segmented and remembered everyday activities as well as young adults^36^. Further, knowledge helps older adults slow down and focus on important event boundary information^54^ while guiding their attention to the most goal-relevant information^55^. Finally, these knowledge-related benefits in encoding partially accounted for the knowledge-related benefits on memory^36^. It follows that increasing an older adult’s semantic knowledge for an activity could aid in their encoding of a related event and improve their long-term memory for the event.

It is important to note that nearly all studies evaluating prior knowledge, event segmentation and memory, including those conducted in our laboratory, have used a quasi-experimental design with naturally occurring groups of experts and novices^43,44,56^ or manipulations of prior knowledge that the participants had acquired over a lifetime (i.e., they came into the experiment with this knowledge)^36,54,55^. However, we know of three prior studies that experimentally manipulated knowledge and evaluated its effects on segmentation ability.

### Prior studies evaluating knowledge-based interventions on segmentation

In the first study, Zacks et al. (Experiments 2 and 3) recruited participants who were unfamiliar with saxophones and randomly assigned them to a trained group (n = 12) or control group (n = 12)^57^. The trained group was given a short course (8 min) on how to assemble a saxophone, and then both groups watched and segmented a video of a female actor assembling a saxophone. Zacks et al. found that training did not affect segmentation behavior. They hypothesized that the target activity may not have been completely novel in that it shares recognizable components of other familiar activities. That is, although people may not have specific knowledge of assembling saxophones, most people have experience assembling other objects (e.g., furniture, toys). Further, they did not include a baseline segmentation measure to evaluate whether the training had any within-subject effect.

In the second study, Blasing used a quasi-experimental design to study the segmentation of dance movements by dancers (n = 10) and non-dancers (n = 12)^42^. They found that dancers segmented significantly less often than did non-dancers. In a second study (a true experiment), dance amateurs (n = 8) spent 6 weeks learning the dance choreography from the video. Segmentation data was collected before and after learning the choreography, and results showed that participants segmented less often after they became familiar with the dance movements. These results provide causal evidence that knowledge influences event segmentation. Unfortunately, however, no control group was included, which makes it difficult to determine whether the true cause of the segmentation changes was due to learning or due to another explanation, such as practice effects.

In the third study, Levine et al. examined how knowledge influences the perception of goal-directed actions^44^. In Experiment 2, they tested ice-skating experts (n = 25), novices (n = 23) and familiarized novices (n = 23) (i.e., participants viewed an ice-skating video once, and then segmented it during their second viewing). They found that all three groups segmented the activity at times when goals were completed and that experts showed higher segmentation agreement than the two novice groups. Importantly, the familiarized novices did not differ from the unfamiliarized novices, which replicates prior work showing that segmentation did not differ based on the number of viewings^46^.

Notably, most of the sample sizes in these experiments were fairly small (ranging from 8 to 25 participants). Additionally, because Zacks et al. did not include a pre-test baseline measure to evaluate within-group changes, they could only evaluate between-group differences^57^. Moreover, Blasing’s Experiment 2 (true experiment) did not include a control group. The only significant changes in segmentation across these three studies were observed with high “doses” of learning (Blasing; 6 weeks of learning the dance phrase)^42^. Finally, these studies only included young adults, who may be more likely to use low-level perceptual features to guide segmentation rather than high-level knowledge structures^36^. Thus, it is possible that a knowledge intervention specifically targeted at older adults may have a stronger effect on event segmentation. It should also be noted that none of these studies evaluated the effect of knowledge on event memory.

### Current study

To address some of these issues in the current study, we experimentally manipulated knowledge by recruiting older adults who were novices in two activities (Tai chi and gyoza making). We then randomly assigned them to workshops designed to increase their knowledge in one activity. Their event segmentation ability and memory performance––measured through an expertise survey, free recall response, recognition memory task, and order memory task––were assessed before and after the workshops. By doing so, we have pre- and post-test assessments in which participants serve as their own control group (trained vs. untrained activity), and we can evaluate both within and between group differences. Further, the intervention consisted of two one-hour workshops. While this “dosage” is not as high as Blasing^42^, it is notably higher than that used by Zacks et al. (eight-minute course)^57^ and Levine et al. (two video viewings)^44^. Our hypotheses, design and analysis plan were pre-registered on Open Science Framework (link in Data availability section). We hypothesized that segmentation agreement and memory would increase following the knowledge-based intervention, and specifically, that segmentation and memory benefits would be primarily found for the trained activity. Results supporting this hypothesis would suggest that a knowledge-based intervention is a viable tool for improving older adults’ episodic memory.

## Results

### Assessment of learning before and after the workshops

To evaluate the amount of information participants learned in the workshops, we compared pre- and post-test scores on an expertise survey. Prior to the workshops, all participants were novices in both activities (expertise survey scores < 10). The survey had a total of nineteen Tai chi questions and twenty gyoza-making questions; however, for these analyses, we removed three qualitative questions from each activity (e.g., “Who does the cooking in your house?”; “How experienced are you with any form of martial arts?”). Thus, we used data from sixteen Tai chi questions and seventeen gyoza-making questions in the following analyses.

**Figure 1:**
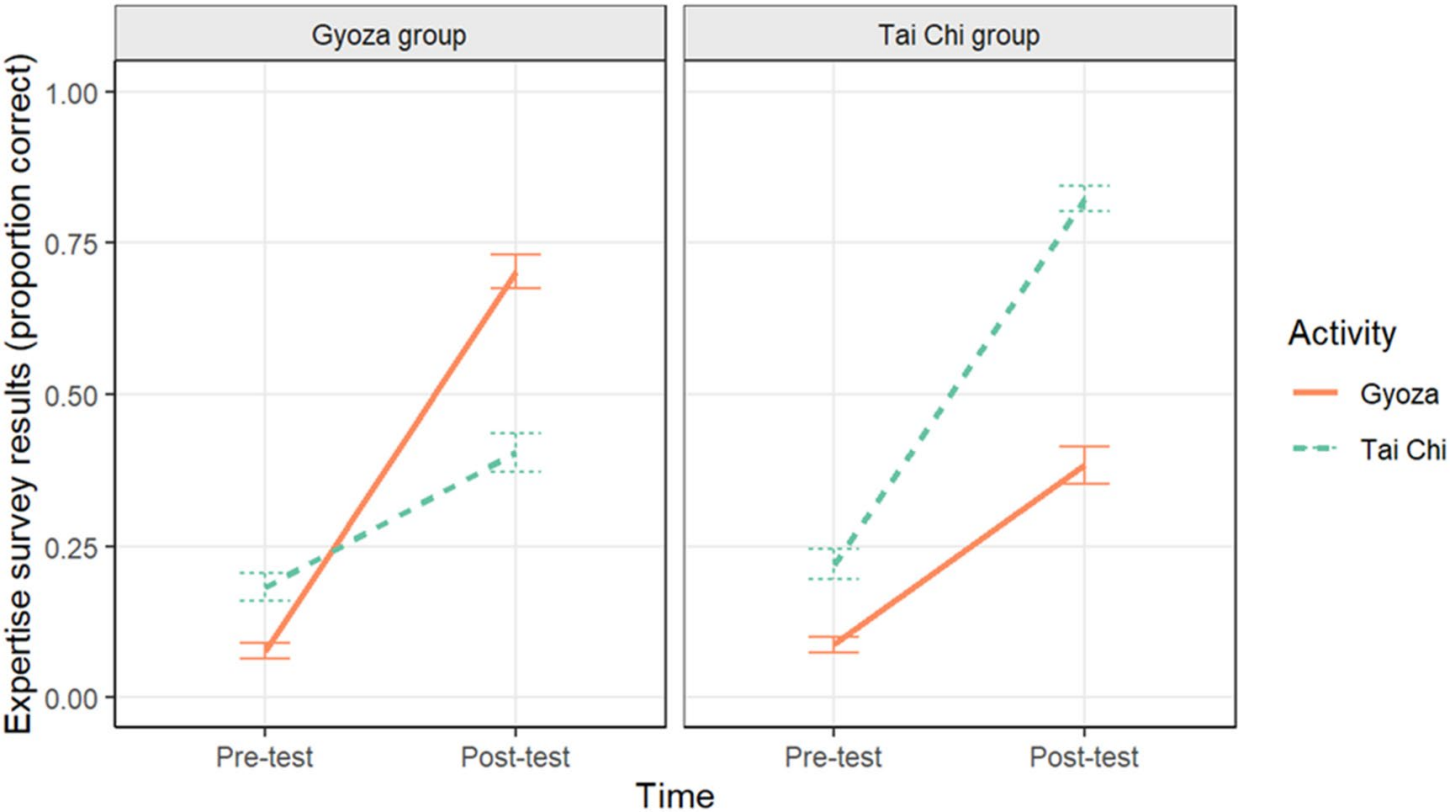
Mean performance on the expertise surveys plotted separately for the Gyoza (left panel) and Tai chi groups (right panel) by activity. Error bars represent one standard error of the mean.

A multilevel binomial logistic regression was conducted with the fixed effects of Group (Tai chi group vs. Gyoza group), Time (pre- vs. post-test), Activity (Tai chi vs. Gyoza), and their interactions and random effects of Participant (intercept effect) and Time (slope effect varying for each participant) predicting the proportion correct on an expertise survey (with the proportion weighted by the total number of questions per activity). The predicted values from this model are in Fig. 1 (plots of the raw data are in Supplemental Materials Fig. S1; Table S1). There was a significant effect of Time, (*B* = − 1.14, *z* = − 24.18,* p* < 0.001), such that survey scores were lower in pre-test than post-test, and a significant effect of Activity, (*B* = − 0.35, *z* = − 9.61, *p* < 0.001). Survey scores for gyoza were lower than the scores for Tai chi. Further, there was a significant Group x Activity interaction (*B* = 0.42, *z* = 11.40, *p* < 0.001) and Time x Activity interaction (*B* = − 0.16, *z* = − 4.46, *p* < 0.001), both of which qualified by a significant Group x Time x Activity interaction (*B* = − 0.39, *z* = − 10.71, *p* < 0.001). The three-way interaction indicates that survey scores significantly improved at post-test, but each group’s scores improved significantly more for the activity on which they were trained. The Tai chi group improved more on the Tai chi survey (pre-test: *M* = 0.22, *SE* = 0.02; post-test: *M* = 0.82, *SE* = 0.02), whereas the Gyoza group improved more on the gyoza survey (pre-test: *M* = 0.07, *SE* = 0.01; post-test: *M* = 0.70, *SE* = 0.03).

### Segmentation count

**Figure 2:**
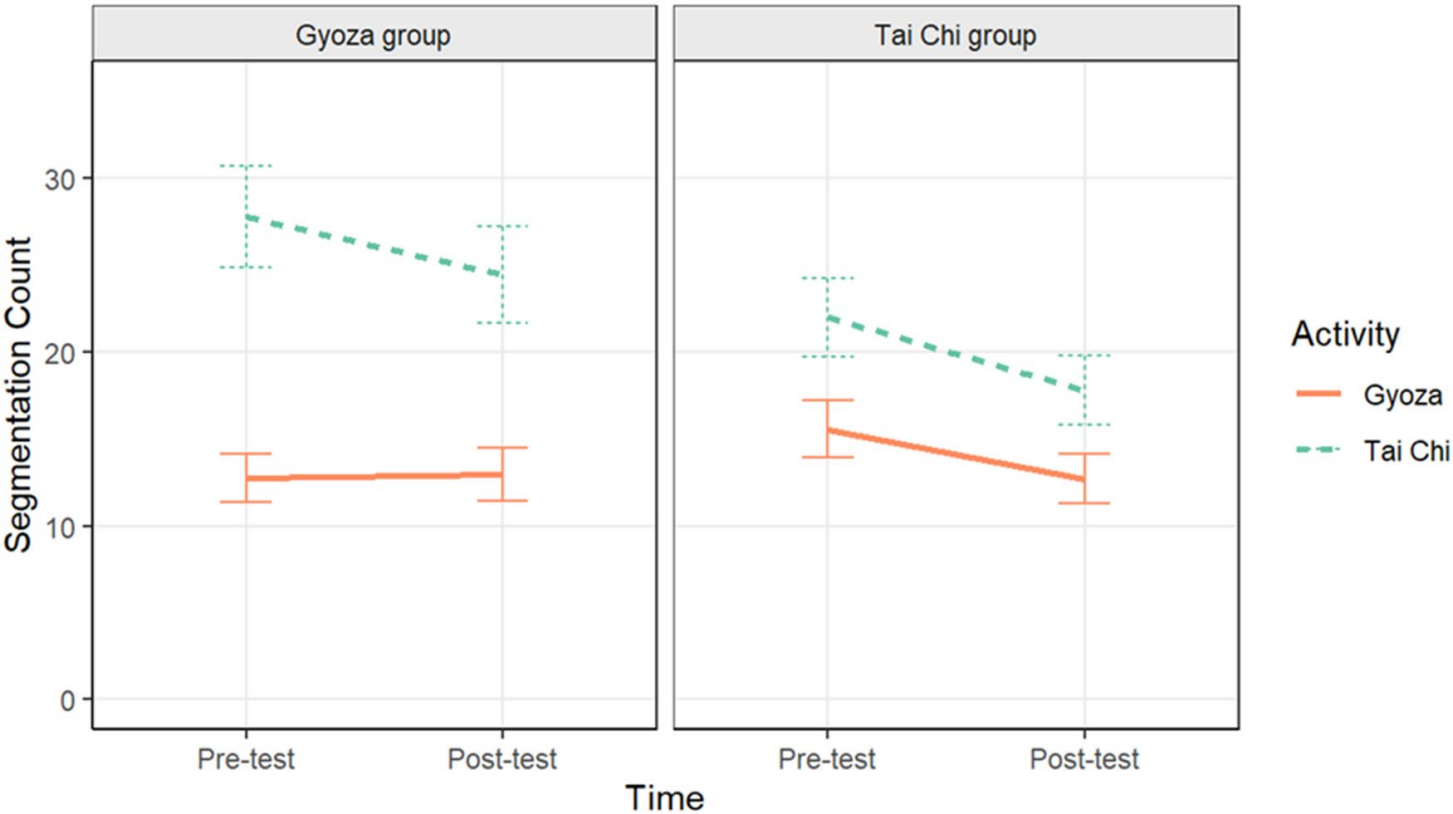
Mean segmentation count (i.e., number of button presses) plotted separately for the Gyoza (left panel) and Tai chi groups (right panel) by activity. Error bars represent one standard error of the mean.

Before evaluating segmentation agreement, we looked at differences in segmentation count, or the number of times participants pressed the spacebar to identify an event boundary throughout the video. Here, we used a multilevel Poisson regression with Group, Time, Activity, and their interactions, along with a random intercept effect of Participant and a random slope effect of Time, to predict the number of times users segmented. The predicted values from this model are plotted in Fig. 2 (plots of the raw data are in Supplemental Materials Fig. S2; Table S2). This regression showed a significant effect of Time (*B* = 0.06, *z* = 1.97, *p* = 0.05), such that participants overall segmented significantly more in pre-test compared to post-test, replicating past work (e.g., Blasing^42^. Note that Fig. 2 depicts a slight increase in segmentation in the gyoza activity from the gyoza group from pre-test to post-test. This increase, from *M* = 12.76 (*SE* = 1.40) to *M* = 12.98 (*SE* = 1.53) is slight and statistically insignificant (*z* = − 0.162, *p* = 0.87). There was also a significant effect of Activity (*B* = − 0.26, *z* = − 20.45, *p* < 0.001), where participants segmented significantly less in the gyoza video than the Tai chi video. Finally, there was a significant Group x Activity interaction (*B* = − 0.09, *z* = − 7.11, *p* < 0.001). In the gyoza video, both groups segmented a similar number of times (Gyoza group: *M* = 12.90, *SE* = 1.29; Tai chi group: *M* = 14.07, *SE* = 1.36; *p* = 0.523) whereas there were larger group differences in the Tai chi video (Gyoza group: *M* = 26.07, *SE* = 2.56; Tai chi group: *M* = 19.80, *SE* = 1.90; *p* = 0.045). However, the other interactions, including the three-way interaction, were not significant.

### Segmentation agreement

**Figure 3:**
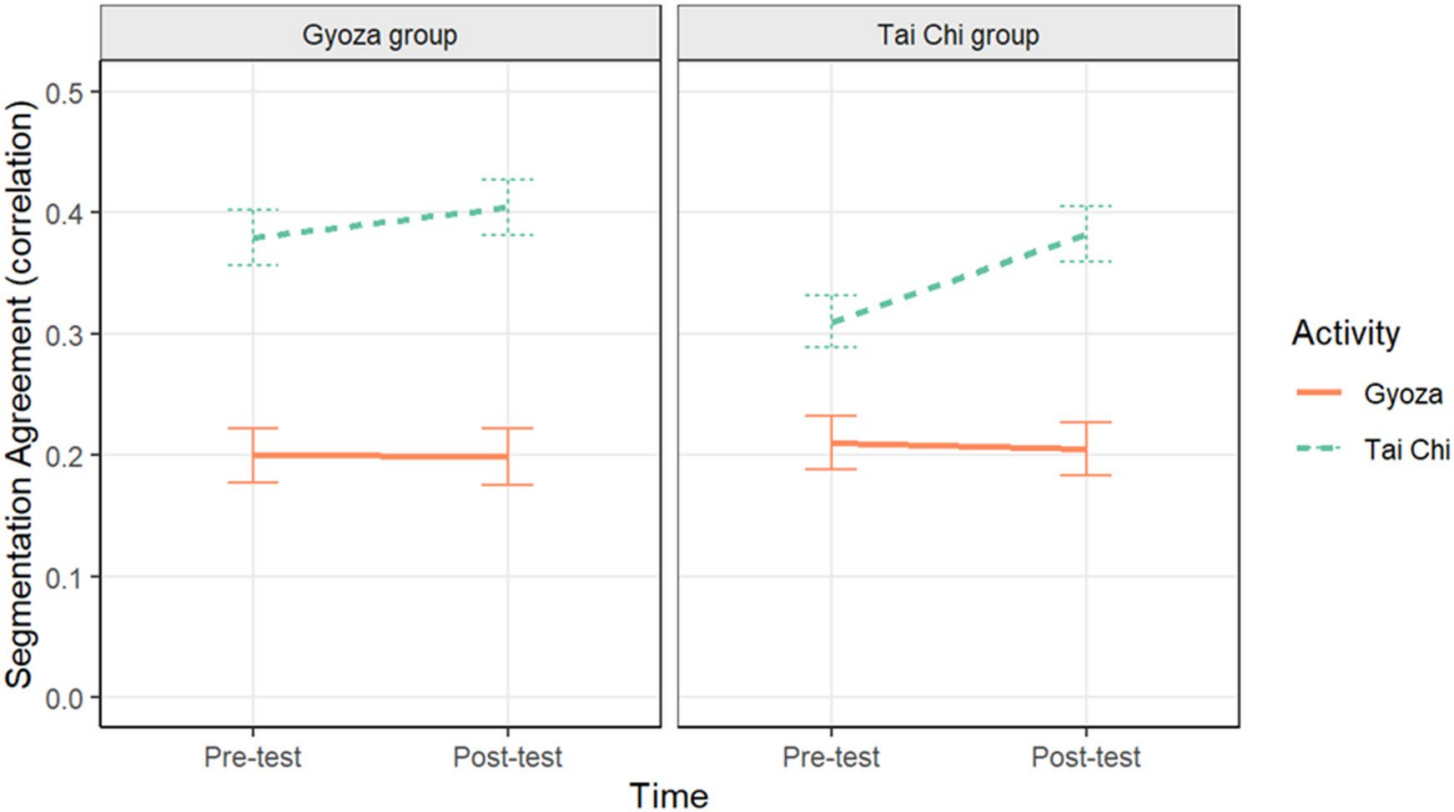
Mean segmentation agreement plotted separately for the Gyoza (left panel) and Tai chi groups (right panel) by activity. Error bars represent one standard error of the mean.

To evaluate our participants’ segmentation behavior, a multilevel linear model was run with Group, Time, Activity, and their interactions, along with Segmentation Count and a random intercept effect of Participant, predicting segmentation agreement. The results of this model are illustrated in Fig. 3 (raw data are plotted in Supplemental Materials Fig. S3; Table S3). This model showed significant effects of Activity (*B* = − 8.27e^−3^, *t* = − 12.25, *p* < 0.001) and segmentation count (*B* = − 3.31e^−3^, *t* = − 10.04, *p* < 0.001). Segmentation agreement was significantly lower for the gyoza video than the Tai chi video, and within both videos segmentation agreement significantly decreased when participants’ segmentation count increased. There were significant interactions between Group and Activity (*B* = − 1.35e^−2^, *t* = − 2.08, *p* = 0.04), and Time and Activity (*B* = 1.29e^−2^, *t* = 2.00,* p* = 0.05). Interestingly, for the Tai chi video, the Tai chi group had significantly lower segmentation agreement (*M* = 0.35, *SE* = 0.02) than did the Gyoza group (*M* = 0.39, *SE* = 0.02). For both groups, segmentation agreement increased for the Tai chi video from pre-test (*M* = 0.34, *SE* = 0.02) to post-test (*M* = 0.39, *SE* = 0.02), and there was no significant improvement for the gyoza video from pre-test (*M* = 0.21, *SE* = 0.02) to post-test (*M* = 0.20, *SE* = 0.02). However, the three-way interaction was not significant.

### Recall

**Figure 4:**
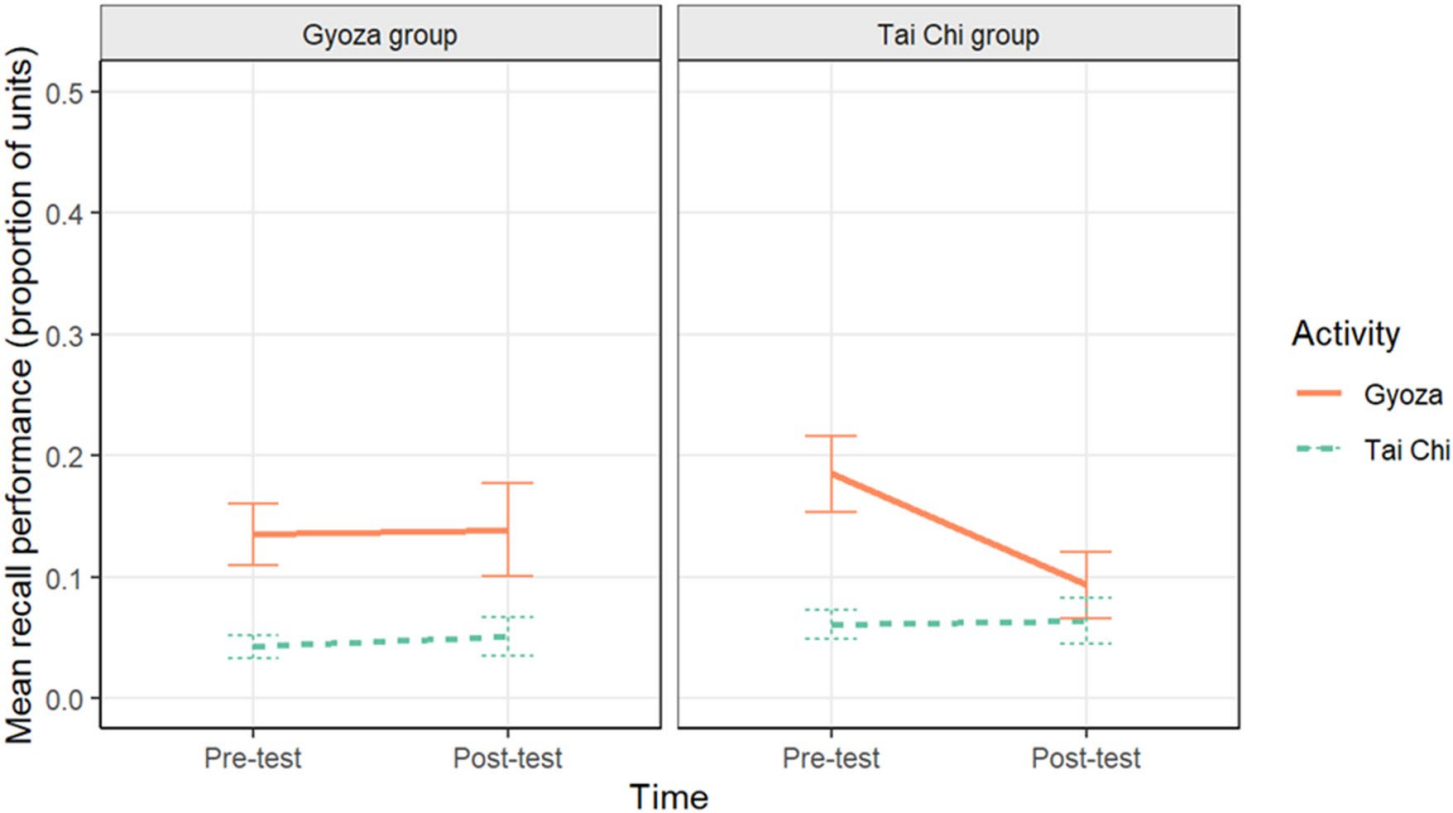
Mean composite recall performance plotted separately for the Gyoza (left panel) and Tai chi groups (right panel) by activity. Error bars represent one standard error of the mean.

To evaluate the effects of the knowledge intervention on recall performance, we ran a multilevel binomial logistic model with Participant as a random intercept effect, and a random slope effect of Time predicting our recall composite variable (average number of A1 and A2 units identified in each free recall response, see Method). In this model, Group, Time, Activity, and their interactions predicted recall. This model (seen in Fig. 4; raw data plotted in Supplemental Materials Fig. S4; Table S4) showed a significant effect of Activity (*B* = 0.50, *z* = 22.64, *p* < 0.001), such that people recalled more from the gyoza video than the Tai chi video. In fact, recall of actions from the Tai chi video was near floor performance. Further, the Group x Activity (*B* = 0.09, *z* = 3.90, *p* < 0.001) and the Time x Activity (*B* = 0.12, *z* = 5.63,* p* < 0.001) interactions were significant as well as the Group x Time x Activity interaction (*B* = − 0.09, *z* = − 3.85, *p* < 0.001). This three-way interaction was mostly driven by a large drop in performance from pre- to post-test in how many actions were recalled about the gyoza video from participants in the Tai chi intervention group (pre-test: *M* = 0.19, *SE* = 0.03; post-test: *M* = 0.09, *SE* = 0.03), rather than intervention-related increases in recall performance.

### Recognition memory

**Figure 5:**
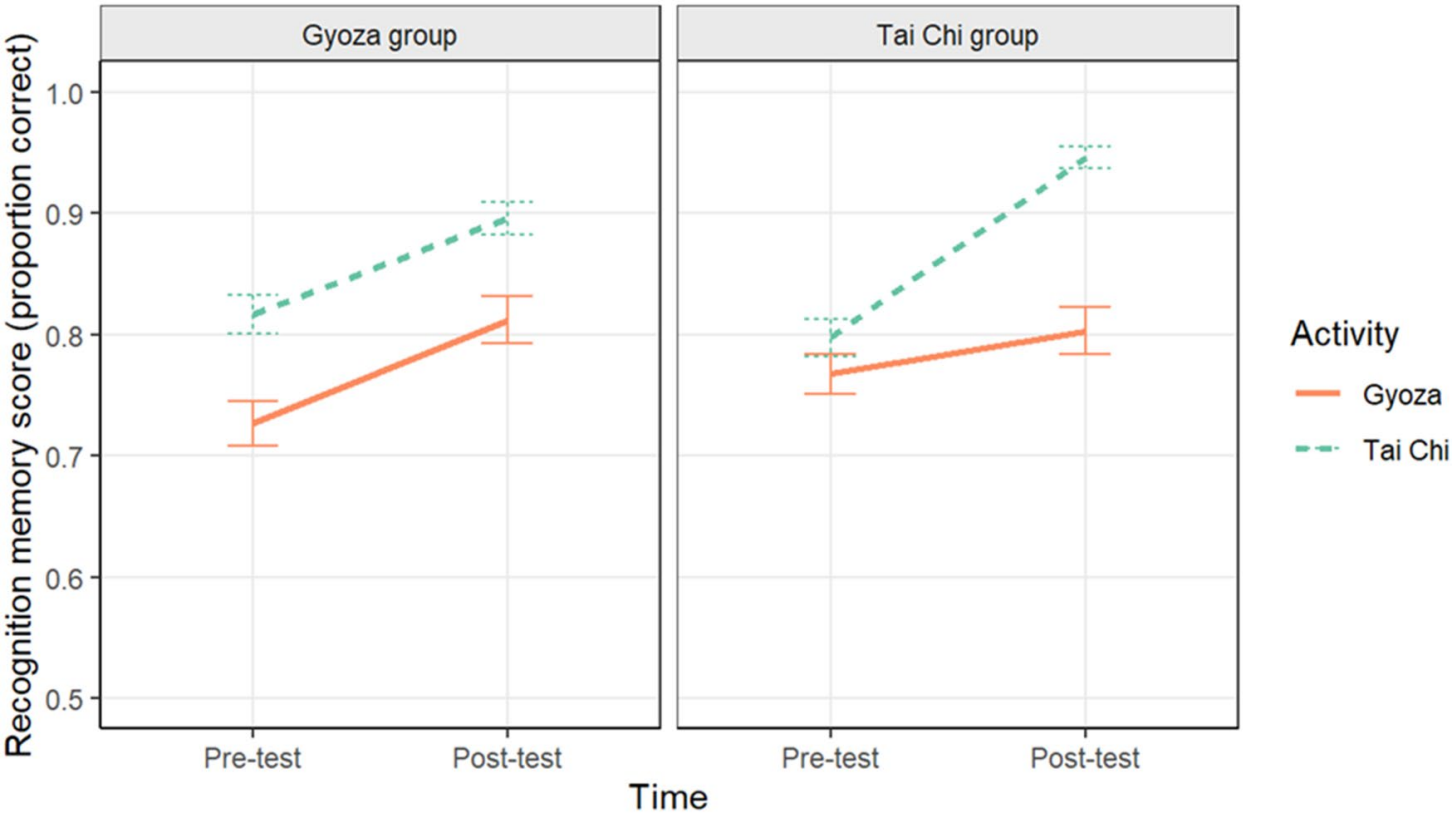
Mean recognition performance plotted separately for the Gyoza (left panel) and Tai chi groups (right panel) by activity. Error bars represent one standard error of the mean.

Recognition memory for the videos watched was tested in a 2AFC task, and performance was calculated as the proportion of correct trials. We ran a multilevel binomial logistic model with Group, Time, Activity, and their interactions predicting recognition performance with a random intercept effect of Participant and a random slope effect of Time. This can be seen in Fig. 5 (raw data are plotted in Supplemental Materials Fig. S5; Table S5).

Recognition was significantly lower for the gyoza video than the Tai chi video (*B* = − 0.25, *z* = − 8.34, *p* < 0.001), and all scores were lower at pre-test than they were at post-test (*B* = − 0.36, *z* = − 8.34, *p* < 0.001). There was also a significant Time x Activity interaction (*B* = 0.18, *z* = 5.15, *p* < 0.001), which was qualified by a significant three-way interaction between Group, Time, and Activity (*B* = − 0.14, *z* = − 3.94, *p* < 0.001). The Gyoza group had a larger increase in recognition performance in the gyoza video from pre-test (*M* = 0.73, *SE* = 0.02) to post-test (*M* = 0.81, *SE* = 0.02) than the Tai chi group did (pre-test: *M* = 0.77, *SE* = 0.02; post-test: *M* = 0.80, *SE* = 0.02). Further, the Tai chi group had a larger increase for the Tai chi video from pre-test (*M* = 0.80, *SE* = 0.02) to post-test (*M* = 0.95, *SE* = 0.01) than the Gyoza group did (pre-test: *M* = 0.82, *SE* = 0.02; post-test *M* = 0.89, *SE* = 0.01).

### Order memory

**Figure 6:**
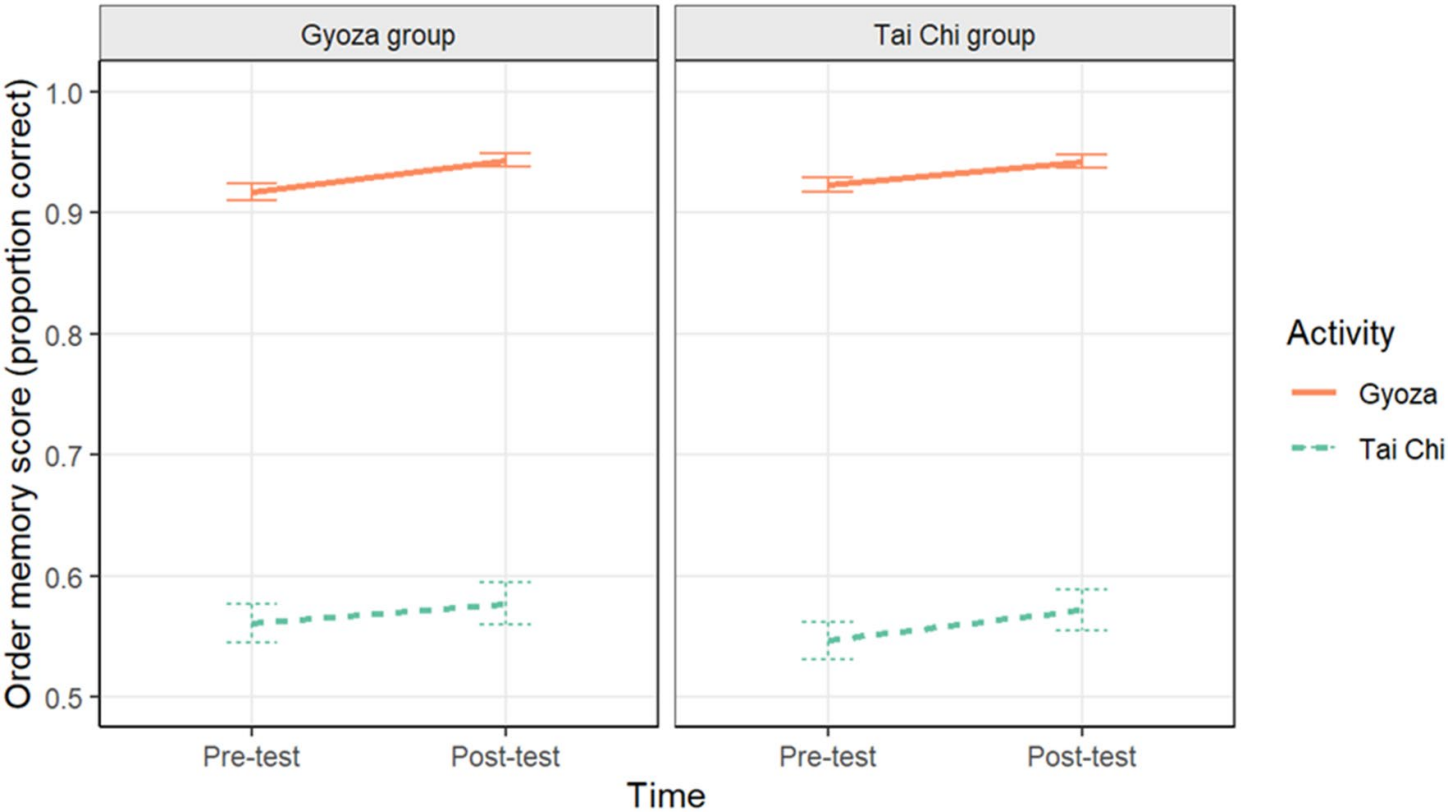
Mean order memory performance plotted separately for the Gyoza (left panel) and Tai chi groups (right panel) by activity. Error bars represent one standard error of the mean.

Memory for the sequential order of actions in the video was also measured using a 2AFC task, and performance was calculated as the proportion of correct trials. We conducted another multilevel binomial logistic model with Group, Time, Activity, and their interactions predicting order memory performance with a random intercept effect of Participant and a random slope effect of Time. This model can be seen in Fig. 6 (raw data are plotted in Supplemental Materials Fig. S6; Table S6). We observed a significant effect of Time (*B* = − 0.11, *z* = − 3.95, *p* < 0.001), with performance significantly lower in pre-test than post-test, and a significant effect of Activity (*B* = 1.18, *z* = 54.09, *p* < 0.001), such that order memory scores were significantly higher for the gyoza video than the Tai chi video. There was a significant Time × Activity interaction (*B* = − 0.07, *z* = − 3.09, *p* = 0.002). Scores increased more for the Tai chi activity from pre-test (*M* = 0.55, *SE* = 0.01) to post-test (*M* = 0.57, *SE* = 0.01) than for the gyoza activity (pre-test: *M* = 0.92, *SE* = 0.004; post-test: *M* = 0.94, *SE* = 0.004). No other effects were significant.

## Discussion

The main goal of the current study was to evaluate whether a knowledge-based intervention would improve older adults’ event segmentation (and encoding of event information), and their memory for everyday events. To do so, we experimentally manipulated semantic knowledge, which has been previously shown to lead to better event segmentation in older adults^36^, through an experiential learning intervention. Older adult novices were randomly assigned into one of two two-hour workshops (gyoza-making or Tai chi) designed to increase their relevant knowledge in one activity. Their event segmentation ability and memory performance were assessed before and after the workshops so that we could evaluate intervention-related gains. We hypothesized that segmentation agreement and memory for the activities would increase following the knowledge-based intervention, specifically within the trained activity.

We found that, following the workshops, older adults’ knowledge—as assessed by the expertise survey—increased for both the trained and untrained activities. Increased knowledge scores for the untrained activity are likely due to practice effects from having completed the expertise survey before, because we observed much stronger learning effects for the trained activity (see Fig. 5). Despite this, we did not observe large increases in segmentation agreement. The Tai chi group’s segmentation agreement for the Tai chi video did increase after the workshops, but the Gyoza group’s agreement for the gyoza video did not. One explanation for this effect is that the Tai chi video may have been easier to segment due to the easily-perceptible bottom-up changes in motion patterns (e.g., arm movement ends, neck movement begins). The perceptual changes associated with Tai chi’s repetitive movements may have signaled to the viewer that an event boundary is occurring, and the viewer may have been more aware of this pattern in the second viewing of the video (i.e., practice effects), regardless of their relevant knowledge about Tai chi. This explanation is supported by improvements in the Gyoza group’s segmentation of the Tai chi video, despite the fact that they did not undergo workshops about this activity. Additionally, this explanation aligns with prior work finding that perceptual features are important to event segmentation^46–49,51^.

Further, the gyoza workshop did not lead to better segmentation for the gyoza activity perhaps because gyoza-making is not as novel to our participants as Tai chi. Although we recruited novices in each activity and people reported having no direct experience making gyozas, most of our participants reported cooking in some capacity (87% report at least taking turns cooking in their home; 42% report specifically using wonton wrappers at least once before). Over time, these cooking experiences create relevant schema and scripts (e.g., gather ingredients, prepare food, cook food) that, on some level, may have facilitated the segmentation and encoding of the gyoza video. This potential issue is similar to that from Zacks et al.: their participants had no direct experience assembling a saxophone, but they have assembled furniture and/or toys, which may explain why the training did not influence segmentation agreement in these activities^57^.

Additionally, it is possible that the constrained nature of our tasks influenced segmentation behavior. Our tasks included a narrow range of possible actions and outcomes and featured a definite end goal (more so gyoza-making than Tai chi). This aligns with the idea that our tasks were more closely related to the saxophone assembly task from Zacks et al. than the dancing or ice-skating activities from the Blasing ^42^ and Levine et al.^44^ studies. Future research may benefit from comparisons of segmentation agreement between constrained and free tasks that have a wider range of actions and no defined end goal. It may be possible that segmentation differences are weaker in constrained tasks.

Interestingly, we did not observe consistent knowledge-related improvement across all memory measures. We did observe the hypothesized patterns of performance in the recognition data such that the Gyoza group improved more on the gyoza recognition task following the workshops, and the Tai chi group improved more on the Tai chi recognition task. The recognition task required specific knowledge about the structure of the activity to garner a correct response because only the action differed (i.e., actor, setting, and props were consistent). Order memory did increase after the workshops, but it was not specific to the trained activity. Surprisingly, we did not see a knowledge-related benefit in recall performance like has been found in prior studies^6,25,58^. Both order memory and recall scores were higher for the gyoza activity at pre- and post-test, which could be attributed to prior cooking knowledge or the relative visual simplicity of the Tai Chi video.

### Limitations

The current study does have limitations. First, this study was completed via online platforms (Pavlovia and Zoom) due to necessary safety precautions during the pandemic. While it provided older adults a great opportunity to engage with others outside of their home, the online platforms may have limited the strength of the intervention “dose” compared to in-person workshop sessions. There was always a researcher present during the Zoom workshop sessions to respond to questions and concerns; however, an in-person workshop would provide more one-on-one attention and guidance to participants. Our workshop instructor was very thorough and attentive, but in-person instruction may have boosted learning more so than online instruction^59^.

Second, we evaluated the extent to which newly-acquired knowledge affects event segmentation behavior. To measure this, we had participants complete an overt segmentation task (i.e., button press when an event is ending and a new event is beginning). However, past work has indicated that overt segmentation tasks may interfere with the ongoing perceptual processes they attempt to measure^29^ and that covert measures of segmentation (e.g., reading time, dwell time, eye movements, brain activity) may be better measures, particularly for older adult participants who may struggle with the dual task nature of the segmentation task (i.e., maintaining the goal of button pressing while trying to learn and remember the ongoing activity)^36^. Third, we only had one trained and one untrained activity per participant, and these activities varied widely in their visual features and higher-level goal structure. To limit the differences amongst videos, we ideally would include multiple activities per condition, but this would have put further strain on the already complicated and laborious research design.

### Future research

More research is needed to evaluate whether prior knowledge plays a causal role in the segmentation and memory organization processes. While some work has found that prior knowledge does influence how people perceive and remember events^36,43,55,56^, this work used quasi-experimental designs. It is important to understand how prior knowledge helps to structure everyday experience in long-term memory, and this is especially true for older adults who experience declines in episodic memory but retain their semantic knowledge.

Further, more work is needed to create or refine interventions that improve event segmentation and memory. While the results of knowledge-based interventions are mixed^42,44,57^, other interventions have shown some promise. For instance, simply asking people to pay attention to the event structure by segmenting an activity improves memory for up to one month compared to passive movie viewing^39^. Other work has shown that highlighting event boundaries via perceptual cues^60^ or commercial breaks^61,62^ can improve memory.

Finally, future studies should examine differences in situations where knowledge improves segmentation ability^36,42,55^ and situations in which there is no top-down effect on segmentation^46–48^. This could be an issue of dosage or an issue of the visual nature of the events used as stimuli. As mentioned above, Tai chi’s repetitive movement pattern may guide participants’ perception of event boundaries, even with no relevant knowledge. Therefore, background knowledge and related schemas may be more beneficial to the perception of more visually-complex events. During such events, prior knowledge may aid in the allocation of attention to the relevant areas that indicate a change in events, instead of other competing visual stimuli.

### Conclusions

In conclusion, we conducted a two-day online workshop designed to increase older adults’ knowledge in one of two activities (gyoza-making or Tai chi). We found clear learning effects in the trained activity and improved ability to recognize information from the trained activity, but the knowledge intervention did not improve participants’ event segmentation ability. The potential effect of dosage and stimulus complexity on these results may be indicative of the nature of event segmentation. The relatively high segmentation agreement found for the Tai chi video suggests that older adults can leverage bottom-up visual features to guide event segmentation in novel tasks, especially when these bottom-up features are simplistic. Additionally, the lack of an increase in segmentation agreement for the gyoza video from pre- to post-test suggests that older adults may utilize pre-existing schemas and scripts for segmenting new activities that are similar to past experiences.

## Methods

### Participants

To our knowledge, no relevant prior studies exist that evaluate the effects of a knowledge intervention on older adults’ segmentation ability. While Blasing reported significant intervention effects in college students (Experiment 2), no effect sizes or measures of variance were reported^42^. Therefore, we were unable to conduct an accurate power analysis. Given that older adults exhibit more variability in segmentation and memory performance, we decided to recruit a much larger sample than that reported in Blasing^42^ (n = 8 per group) to detect effects of knowledge on segmentation within older adults. Study procedures were approved by Kansas State’s Institutional Review Board. All methods were performed in accordance with the relevant guidelines and regulations. Informed consent was obtained from all subjects prior to their participation.

Participants aged 65–85 years were recruited from an online database maintained by the Memory and Aging lab at Kansas State University as well as through various local and national volunteer organizations. A total of 80 older adults (56 women, 24 men) participated with an overall average age of 71 years (*SD* = 5.4). Recruitment and data collection for this study took place in 2021. Given that this was an online study, participants were recruited from across the country (i.e., Kansas, Missouri, Texas, Ohio, Illinois, New York).

Table 1 contains the demographics of each group. Independent-samples *t*-tests with equal variance compared the two groups for each of the continuous variables (age, MoCA score, and education in years). Semantic fluency was calculated from the number of unique items produced in two measures (category and letter fluency) given during the pre- and post-intervention tests (see section “Semantic Fluency” below). No significant difference between groups was found.

**Table 1:** Demographic information by workshop group.

	Gyoza group (*n* = 38)	Tai chi group (*n* = 42)	Independent samples *t*-test
Gender	24 females/14 males 63.15% Females	32 females/10 males 76.2% Females	
Racial distributions	37 white, 1 more than one race	39 white, 1 Asian, 1 more than one race, 1 prefer not to answer	
Age	71.00 (*SD* = 4.74)	71.00 (*SD* = 6.06)	*t*(78) = 0.00, *p* = 1.00
MoCA	13.23 (*SD* = 1.30)	13.61 (*SD* = 1.20)	*t*(78) = − 1.36, *p* = 0.17
Education	16.55 (*SD* = 2.85)	16.8 (*SD* = 2.93)	*t*(78) = − 0.39, *p* = 0.69
Semantic fluency	42.20 (*SD* = 9.20)	42.02 (*SD* = 10.02)	*t*(153) = 0.11, *p* = 0.91

Education = average number of years of education; MoCA = average score on the mini-MoCA test; Semantic fluency = average number of items produced on category and letter fluency measures (measured at pre-test and post-test).

**Figure 7:**
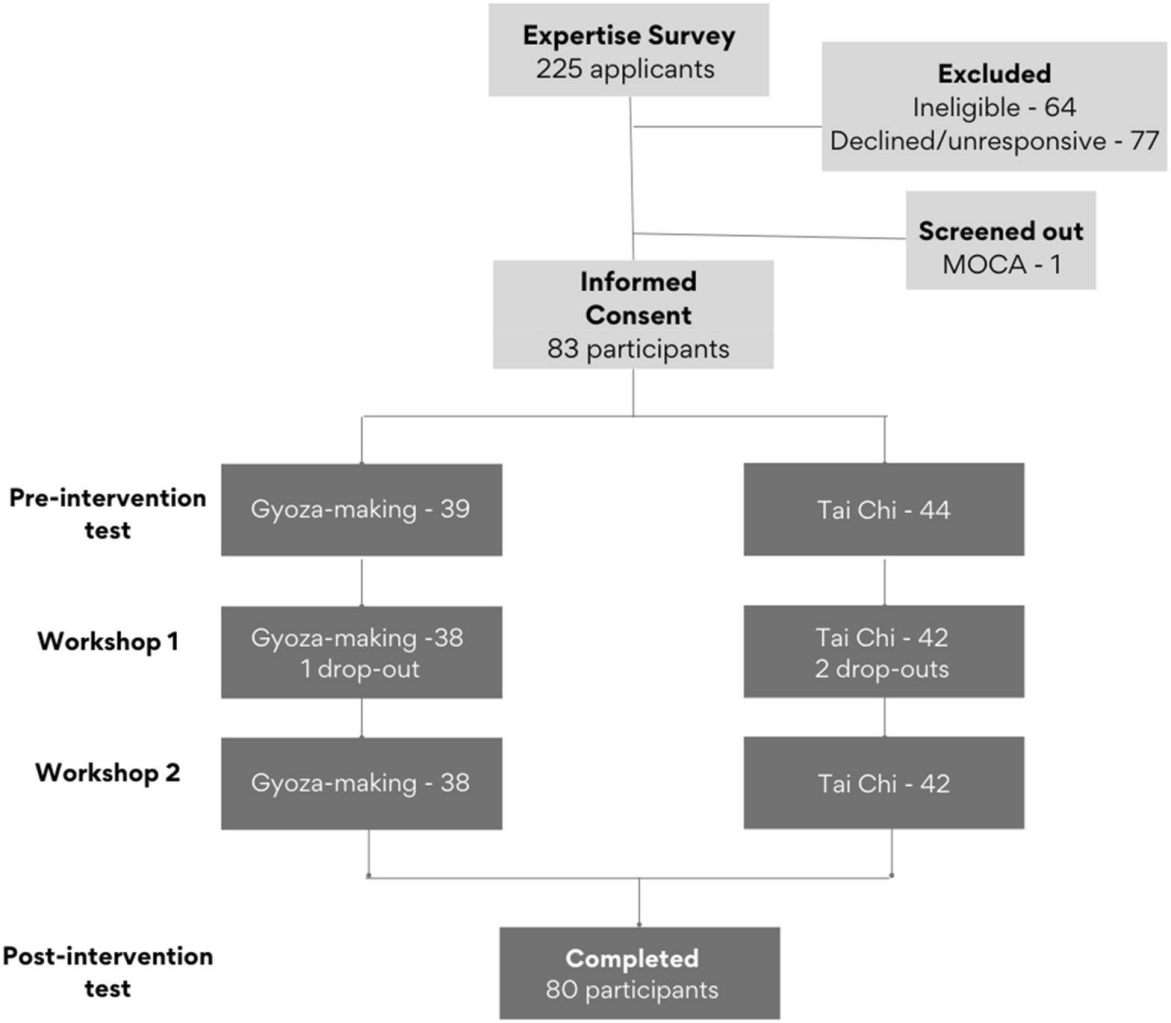
Flow of participants through the experimental design.

Figure 7 is a schematic for the recruitment numbers and the experimental design. To be eligible for the study, potential participants had to complete an expertise survey that tested knowledge of two activities: Tai chi, a low-impact Chinese martial art, and gyoza-making, preparing Japanese dumplings (see Supplemental Materials S1).

The survey was designed by the expert responsible for instructing each workshop. It was composed of nineteen (Tai chi) and twenty (gyoza-making) multiple choice questions, which were based on the materials that were going to be covered during the training workshops. No feedback on the scores was provided to participants regarding correct answers nor their scores. All topics in the expertise survey were taught during the respective workshops. Participants would then retake the expertise survey during the last portion of this study to evaluate learning from the workshops.

Two-hundred and twenty-five participants completed the survey but sixty-four of those scored higher than our cutoff score (> 10 correct answers for each activity).They were deemed too knowledgeable and were excluded. Participants that scored less than 60% correct in a test were deemed novices.

If a potential participant was deemed a novice in both activities, a research assistant scheduled a phone call to determine whether the participant was cognitively healthy and physically able to participate in the activities. The phone screening lasted approximately thirty minutes and covered questions related to demographics, medical history, contact information, and two brief dementia screening measures: AD8 (score > 2 = ineligible)^63^ and Short Blessed (score 0–4 is healthy cognition; > 10 = ineligible)^64^. In addition to being novices in the targeted activities, the eligibility requirements excluded people with significant visual problems, enduring neurological problems, unmedicated psychiatric disorders, excessive drinking, use of benzodiazepines and other memory-interfering drugs. Additionally, only fluent English speakers were included to ensure comprehension of the verbal memory tests and communication during workshops. Finally, to evaluate overall cognitive functioning, the participants completed the brief version of the Montreal Cognitive Assessment (Mini-MoCA)^65^ via Zoom platform. This version of the MoCA was selected due to its reliability for remote screening of cognition. Prior to using Zoom, participants were offered Zoom training, ensuring that each participant felt comfortable with the platform. Participants were scored by a certified MoCA rater using the official instructions for the Mini-MoCA version 2.1. Participants were not informed of their score during the screening process and were allowed to participate in the workshops regardless of their scores. However, only participants with a score of eleven or above (out of fifteen) were officially included in the data analysis of this study, which is considered normal cognition (reference MOCA official instructions version 2.1). One person assigned to the Gyoza group was excluded from the data analyses due to an insufficient MoCA score, but they completed participation. Participants were compensated $50 for their participation.

### Materials

#### Videos

Participants were first asked to watch a practice video of a person building a boat from Duplo blocks (duration = 155 s) to become familiar with the experimental procedure. Then they watched two additional videos that were filmed from a fixed perspective without zoom and without audio. Each video contained one actor performing one activity. One video was of an actor performing Tai chi movements (duration = 307 s) and the other video was of an actor making gyozas (duration = 411 s; see Supplemental Materials Fig. S7 for stills from both videos).

#### Segmentation task

We used the unitization task originally reported by Newtson^66^ to assess participants’ event segmentation ability. While watching the videos, participants were asked to press their keyboard’s spacebar each time they believed one natural and meaningful unit of activity in a video ended and another one began (as was done in Zacks et al.^57^). Participants practiced the segmentation task during the practice video described above. To shape participants’ segmentation behavior by helping them focus on events of a similar grain size across the sample, participants needed to segment the practice video at least three times. Participants were not aware of this value. Those who segmented fewer than three times were simply asked to identify a few more units of activity, and the practice video was repeated until they segmented three or more times. Once they met these requirements, the program moved on to the segmentation task for the two experimental videos.

The number of meaningful boundaries identified by a participant (i.e., segmentation count), operationalized as the number of times they pressed the keyboard’s spacebar during a video, was recorded. This value was used as a predictor variable, to evaluate whether the number of perceived events influenced participants’ segmentation agreement and memory performance.

Segmentation agreement is the degree to which one participant’s identified event boundaries align with those identified by others. In our analyses, we used each activity’s knowledgeable group at post-test (those who went through the related workshop) as the reference group to which all other segmentation similarity values would be compared. This is because the knowledgeable groups for each activity should have the most informed normative event boundaries. In other words, for the Tai chi video, each participant’s event boundaries at pre- and post-test were compared with the boundaries identified in the post-test by those who completed the Tai chi workshop. For the gyoza video, individual’s event boundaries at pre- and post-test were compared to the boundaries identified by the Gyoza group at post-test. This was done so that all segmentation agreement values would be calculated in terms of the same reference point.

Segmentation agreement was calculated by fitting a one-second Gaussian Kernel function around each participant’s button press (i.e., each perceived event boundary). By doing so, each frame of the video receives a smoothed likelihood (on a scale of zero to one) that the participant perceived an event boundary on that frame. Normative event boundaries were identified by averaging the event boundary probabilities from each “knowledgeable” participant on the post-test segmentation task. The correlation between each participant’s event boundary probabilities and the knowledgeable group’s normative boundaries was then calculated. Importantly, this was calculated for each video using a leave-one-out procedure––i.e., a participant’s own post-test segmentation responses were not included in the normative boundaries calculated for their comparison. For more on this segmentation agreement calculation, see Pitts et al.^36^ and Newberry et al.^43^.

#### Free recall task

Participants were instructed to recall the video they had just watched and type out a description of that video. No other prompts were provided. This typed free recall procedure has been used with older adults in previous studies^34,36,54^. Free recall performance was scored using the Action Coding System (ACS)^34,36,54,67^. The ACS provides a way of coding free recall of complex activities by grouping actions into larger goals called *A2 units* (e.g., assembles gyozas) and smaller subgoals called *A1 units* (e.g., picks up one wrapper, puts spoonful of food onto the wrapper, moistens edge of wrapper, folds wrapper around food, pleats wrapper edges)*.* The gyoza video consisted of 16 A2 units and 86 A1 units, whereas the Tai chi video consisted of 19 A2 units and 159 A1 units. To score the data, two trained, independent raters both scored the recall data from 3 participants and their reliability was calculated (for A1 units, the average inter-rater Kappa = 0.77; for A2 units, Kappa = 0.88). Discrepancies were discussed and resolved. Then, the raters were assigned to score all of the data for a particular video. Performance was scored as the proportion of action units correctly recalled (i.e., total action units correctly recalled divided by total action units in the video) for both A1 and A2 units. These proportions were strongly correlated (*r* = 0.84, see Table S7 in Supplemental Materials S1 for correlation table of all memory measures), so we averaged these values and created a composite recall variable. Thus, each participant received an average proportion correct for recall performance.

#### Recognition task

The recognition task consisted of twenty two-alternative forced choice (2AFC) trials (see Supplemental Materials Fig. S8 for visualization). For each trial, the participants saw two images: One of these images came from the video the participant just watched, and the other image came from a different video filmed in the same location with the same actor. The participants were instructed to click on the image that came from the video they had just watched. Recognition performance was scored as the proportion of correctly identified images. This 2AFC discrimination between real and foil video stills has been used previously as a measure of event memory^35,39^, including usage with older adult participants^34,36,54^.

#### Order memory task

For this task, participants were given sixty-six 2AFC trials (see Supplemental Materials Fig. S8 for visualization). However, this time both of the images came from the video the participant had watched and the participant was instructed to identify which image came first in the video. Order memory performance was scored as the proportion of correctly identified images. This 2AFC task is a modified version of the order memory task used by Zacks et al.^35^. This modified version has been used previously with older adults by Smith et al.^54^ and Pitts et al.^36^.

#### Semantic fluency tasks

Participants also completed two semantic fluency measures in the pre- and post-intervention tests. In the category fluency measure, they were asked to type as many objects of a certain type as they could in sixty seconds (“animals” in pre-test and “vegetables” in post-test). In the letter fluency measure, they typed as many words starting with a certain letter as they could in sixty seconds (“S” in pre-test and “M” in post-test). Order of the type of fluency test was counterbalanced across participants. Performance was calculated as the total number of unique items produced. This task served as a distractor task that separated the video watching and memory measure (free recall, recognition, and order) stages of the experiment. It also served as a measure to ensure that one workshop group did not excel more than the other in semantic fluency or typing ability, both of which could impact the evaluation of the free recall responses.

#### Knowledge training workshops

To provide participants with knowledge and experiential learning of an activity, they took part in a two-session workshop. Each session was one hour long and took place two days apart (e.g., Tuesday and Thursday of the same week) to prevent fatigue. A script was developed for the gyoza and Tai chi workshops based on their corresponding expertise survey to guarantee inclusion of all relevant information. All script information fit in the one-hour workshop; therefore, participants had the opportunity to learn the material twice during the training.

The workshops took place online via the Zoom platform with a live instructor that provided feedback to participants as they were completing their training. Participants completed the workshops in small groups ranging from one to six people. Group size was based on a participant’s availability and capped at six per group so that the workshop instructor and research assistant were able to closely monitor all participants. The research assistant was also responsible for monitoring participant’s cameras and connection to ensure that they were present and effectively connected to the Zoom meeting.

##### Gyoza workshops

The gyoza workshops started with an introduction to the activity, which covered gyoza origins, similar foods from other cultures and traditional ingredients. It also included specific details such as wrapper thickness, amount of filling, and number of pleats in each gyoza. Prior to the workshops, participants either bought wrappers or made their own, and they also made a filling of their preference. The instructor demonstrated how to make your own wrappers, and how to properly assemble each gyoza. Then participants were given the opportunity to practice assembling their own gyoza. After making a batch, the instructor would switch to the cooking portion of the workshop, which happened during both sessions. Although all participants were required to practice assembling gyozas, they were not required to cook them since some did not have access to a full kitchen when participating in the workshops.

##### Tai chi workshops

The Tai chi workshop also started with an introduction to the activity, including its history, health benefits, and key components of this type of martial art. Participants were then taught a sequence of Tai chi moves that could be done either standing or sitting, depending on the participant’s preference. The sequence included seven different movements performed three times on each side of the body, moving from head to toe. In each workshop, the instructor demonstrated the complete sequence three times with participants following along with the moves.

### Procedure

Due to the COVID-19 pandemic, this entire study was conducted online. If participants met all eligibility criteria described above, they were sent the informed consent form to read and sign. On the consent form, participants were able to indicate whether or not they were familiar with Zoom. Participants that reported they were not familiar with the video conferencing platform were offered training on how to install and utilize the software. Then, to guarantee that all participants would be able to connect to the online workshops and use the conferencing platform’s basic functions, the mini-MoCA assessment was conducted via Zoom after an effective connection was established (audio and video functioning). Despite these measures to ensure technical success, there were two participants who chose to discontinue the study due to connection or other technology-related difficulties (see Fig. 7).

After completing the mini-MoCA, participants received a schedule of their study sessions through an email, including links to Pavlovia.org, the platform used to host the pre- and post-tests, along with the Zoom links for the two workshops. Utilizing the Pavlovia platform required no additional training from participants. After clicking on the Pavlovia link the experiment would begin and instructions for the segmentation and memory tasks would be provided on-screen, just as they would be in an in-person study. During the pre-test, participants watched and segmented the practice video followed by the two experimental video blocks. To proceed to the experimental video blocks, participants had to segment the practice video using at least three segments. In each video block, they watched and segmented a video (e.g., Tai chi or gyoza video), and then they completed one of the semantic fluency tasks as a task separating the video presentation from the memory measures. Finally, they completed the memory measures in this order: free recall, recognition, and order memory. After completing the memory measures, participants moved on to the second video block. The order of videos and order of the semantic fluency tasks was counterbalanced across participants and sessions. Participants completed their pre-tests between four to six days prior to the workshops.

Participants were then randomly assigned to one arm of the study (gyoza-making or Tai chi). One participant dropped out of the study after group assignment because they preferred the other activity (see Fig. 7). The remaining participants took part in two workshop sessions, separated by one day, on Zoom. Between one to two days after the final workshop session, they completed their post-tests on Pavlovia.org. The procedure was the same; however, video order was counterbalanced (if they saw gyoza-making first during the pre-test, they saw Tai chi first during the post-test). At the end of the post-test, participants completed the same expertise survey questions that they were given prior to enrolling in the study to assess how much semantic learning occurred within the workshops. Finally, the participants were debriefed and compensated for their time.

### Analyses

For all analyses, we conducted multilevel models that incorporate both fixed effects of Group (Tai chi group vs. Gyoza group), Time (pre- vs. post-test) and Activity (Tai chi vs. Gyoza) as well as random effects (detailed below). These analyses were conducted in R (version 4.2.1) using the lme4 library^68^ and the emmeans library^69^ was used to calculate the least-squared means for each model. The random effect structure of each model was independently chosen as that which produced the best fitting model (identified as the model with the lowest Akaike Information Criterion value^70^). Further, each multilevel model used a different distribution based on the type of data: Poisson distribution used for count data (e.g., segmentation count), binomial distribution used for binomial data (survey data, all memory measures) and linear distribution used for data with linear relationships (segmentation agreement). All binomial logistic regressions were run with proportions as the predicted variable, using a weight value to inform the models of how many trials there were in total.

Of the tested random effect structures, participant was included as a potential intercept effect (reflecting the random variability of each participant sampled from the population), and Time was allowed to vary as a random slope effect (as each participant could have a unique change in slope over time).

### Supplementary Information

Supplementary Information.

## Data Availability

The de-identified data along with the analytic code and materials for this study are all available on our Open Science Framework (OSF) page (https://osf.io/kr382/?view_only=96cac21556984b8b90481996fdf231d5). Our hypotheses, design and analysis plan were pre-registered on Open Science Framework (https://osf.io/exdk5).

## Code availability

The de-identified data, analytic code, and materials for this study are all available on our Open Science Framework (OSF) page (link in Data Availability section). The analyses were conducted in R (version 4.2.1), using the lme4 library (version 1.1-30) and the emmeans library (version 1.8.0). The study design, hypotheses, and analytic plan were all preregistered on OSF. Sample size determination, data exclusions, manipulations, and measures are detailed below.
