# Supplementary material for: Knowledge-based intervention improves older adult recognition memory for novel activity, but not event segmentation or temporal order memory
Source: Sci Rep. 2023 Oct 31;13:18679. doi: 10.1038/s41598-023-45577-3 (PMC10618285; doi:10.1038/s41598-023-45577-3)
Supplement: Supplementary file 1 — Supplementary Information. [file 41598_2023_45577_MOESM1_ESM.docx]

**Supplemental Materials**

A.


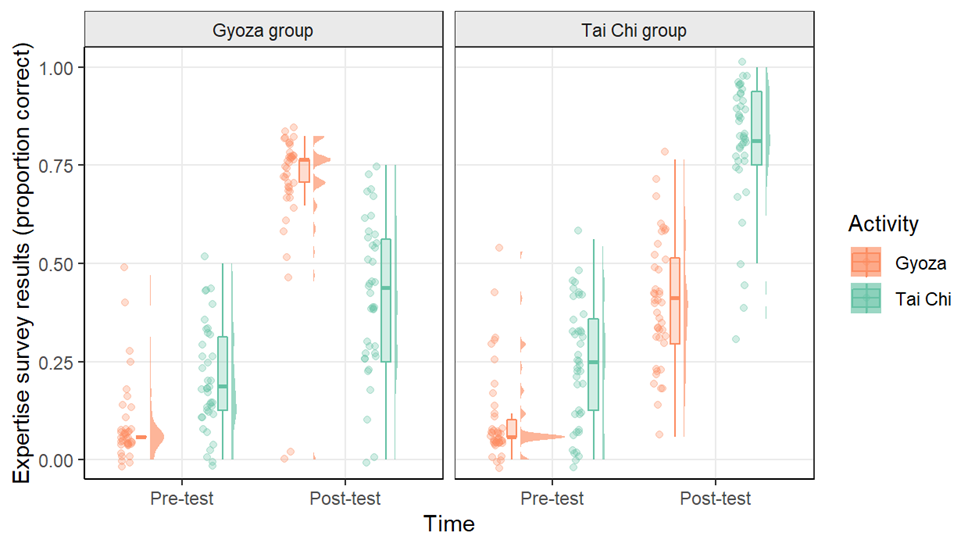


B.


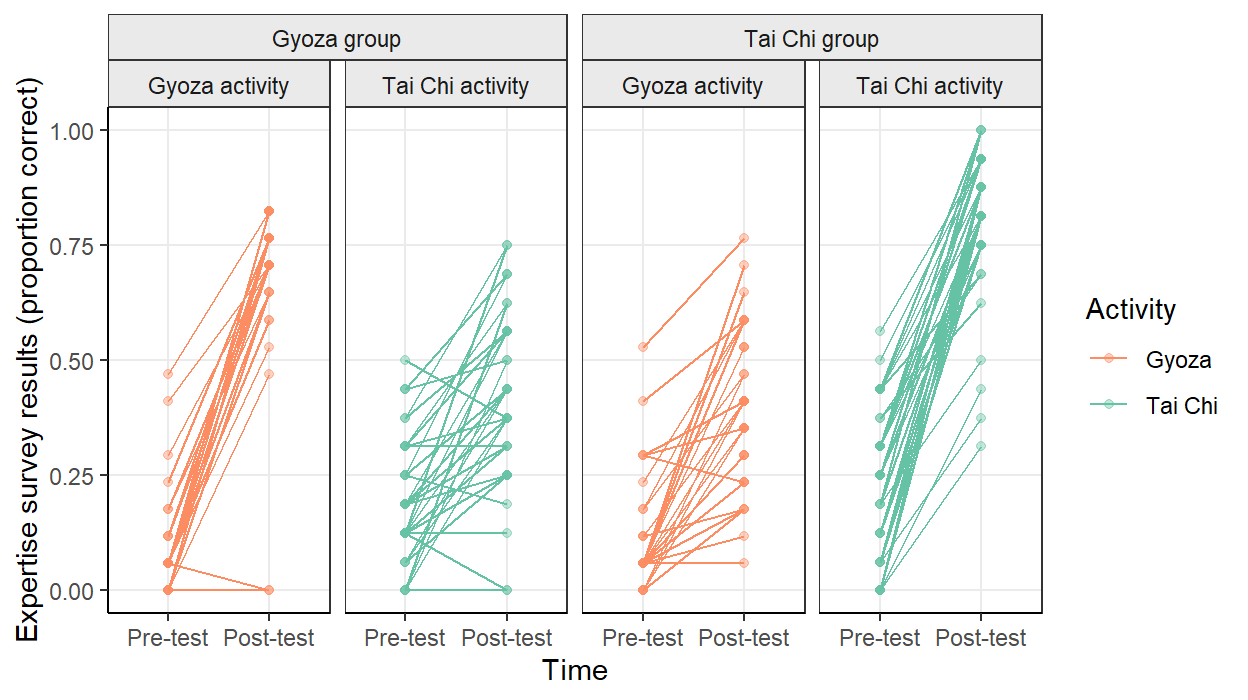


*Figure* S1. Raw data for expertise survey performance in terms of proportion correct. A: Dots represent individual data points; box charts and half-violin plots illustrate the spread of the raw data. B: Lines connect data from the same participant.

A.


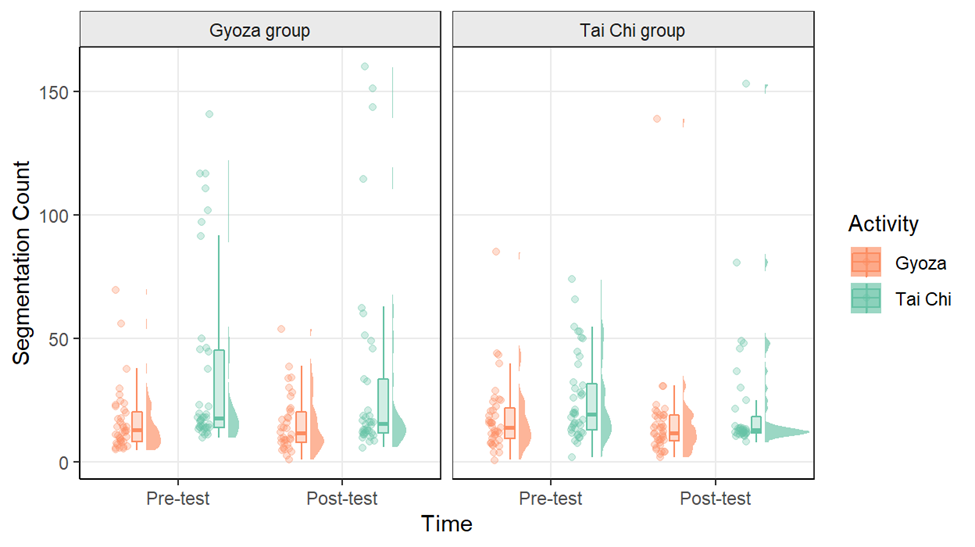


B.


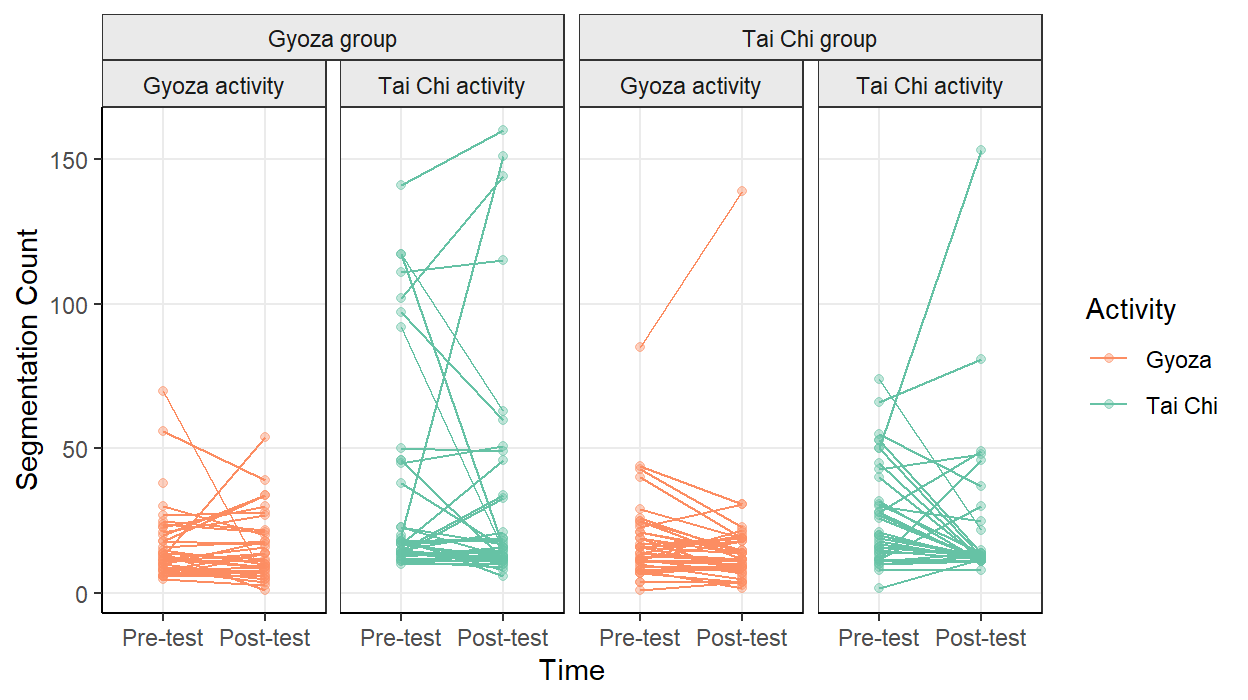


*Figure* S2. Raw data for the number of times participants identified event boundaries within the tested videos. A: Dots represent individual data points; box charts and half-violin plots illustrate the spread of the raw data. B: Lines connect data from the same participant.

A.


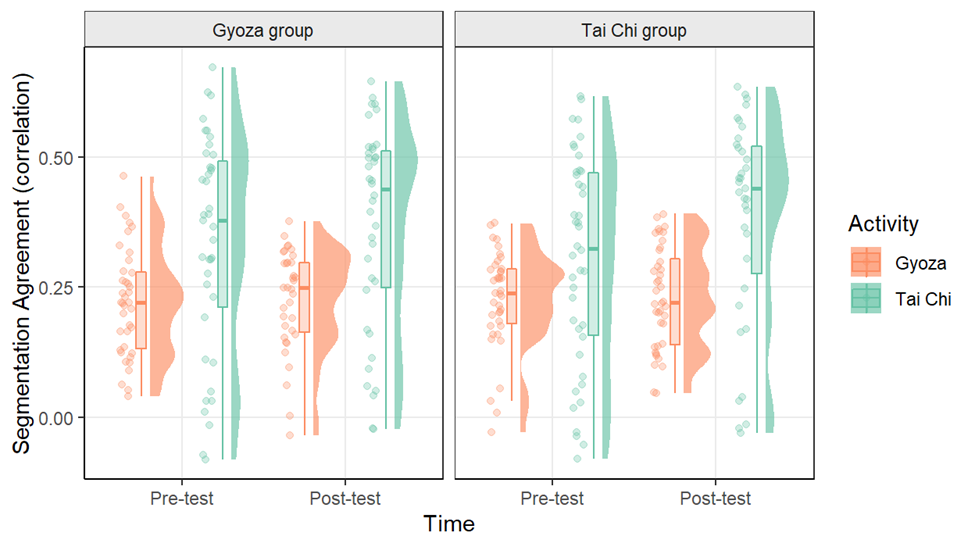


B.


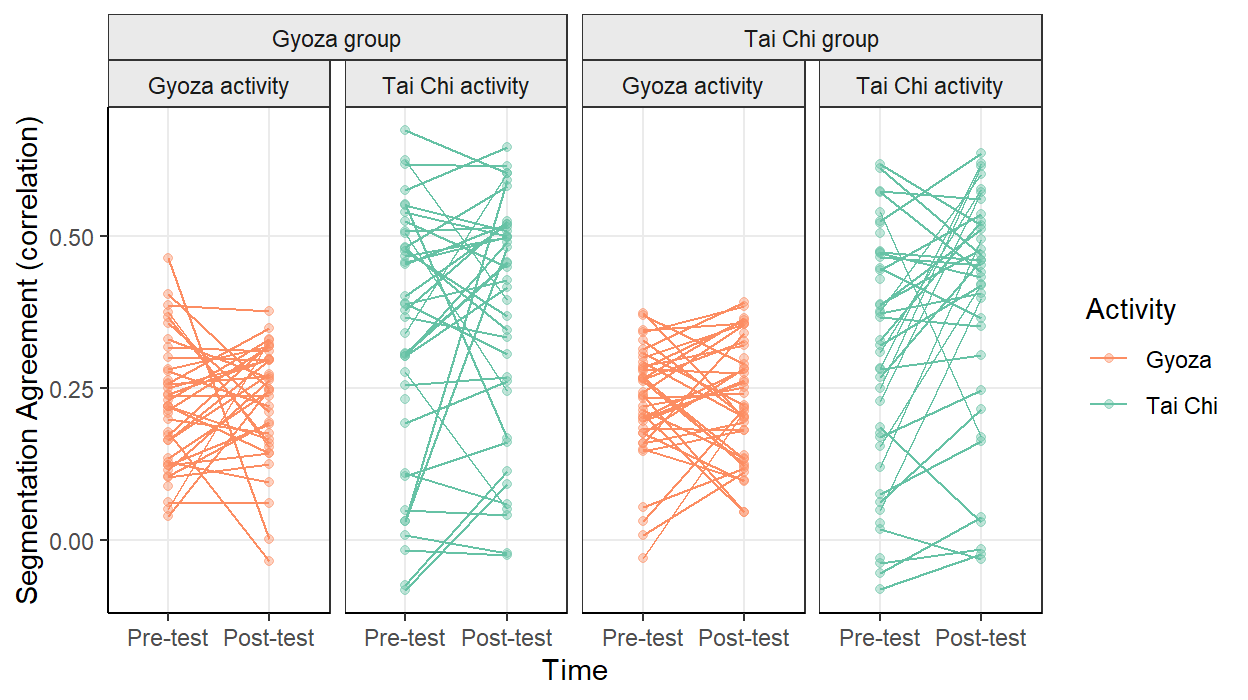


*Figure* S3. Raw data for each participant’s segmentation agreement when correlated with the data from the knowledgeable group at post-test. For the gyoza video, this knowledgeable group is comprised of the individuals who participated in the gyoza workshop. For the Tai chi group, the knowledgeable group is all participants who participated in the Tai chi workshop. A: Dots represent individual data points; box charts and half-violin plots illustrate the spread of the raw data. B: Lines connect data from the same participant.

A.


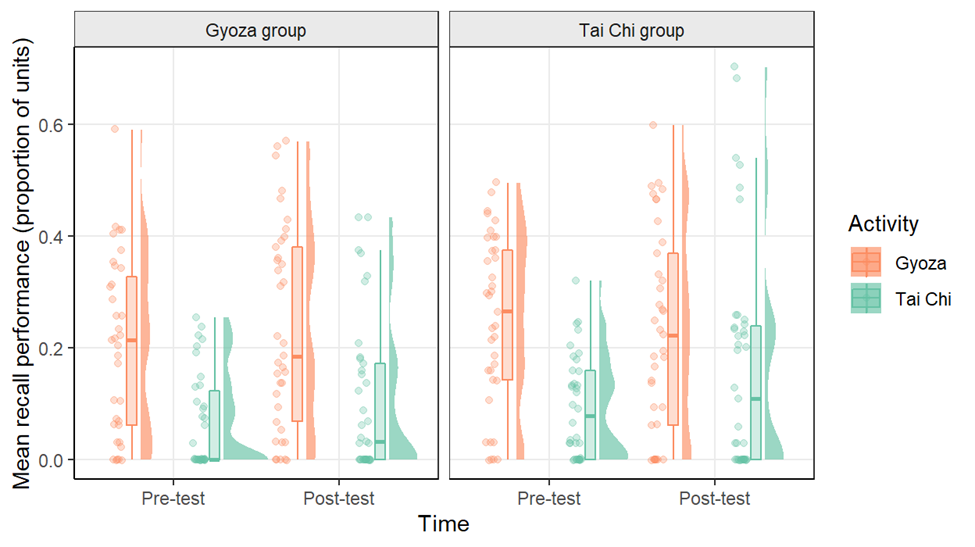


B.


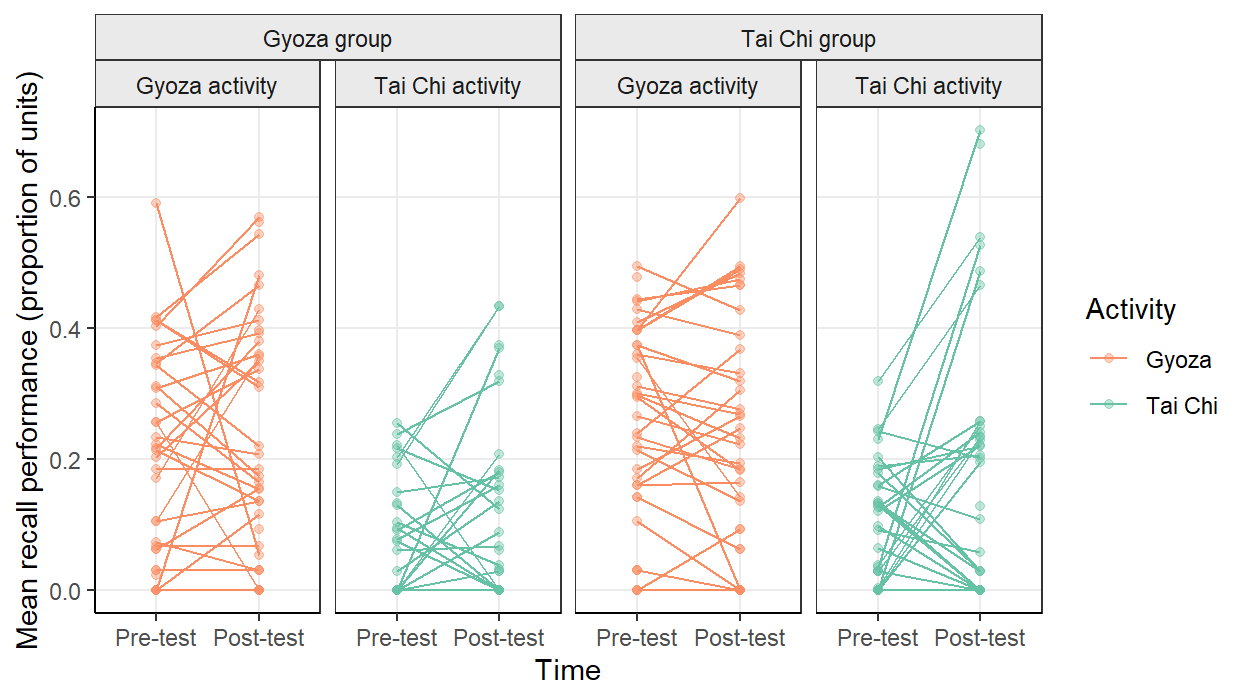


*Figure* S4. Raw data for mean recall scores (averaged between A1 and A2 units mentioned in free response answers), measured as a proportion of total possible units included. A: Dots represent individual data points; box charts and half-violin plots illustrate the spread of the raw data. B: Lines connect data from the same participant.

A.
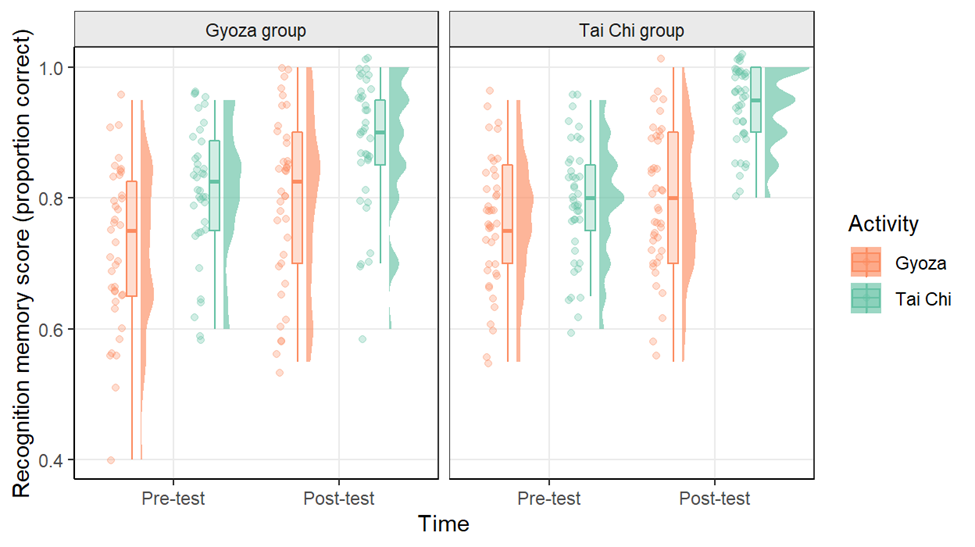


B.


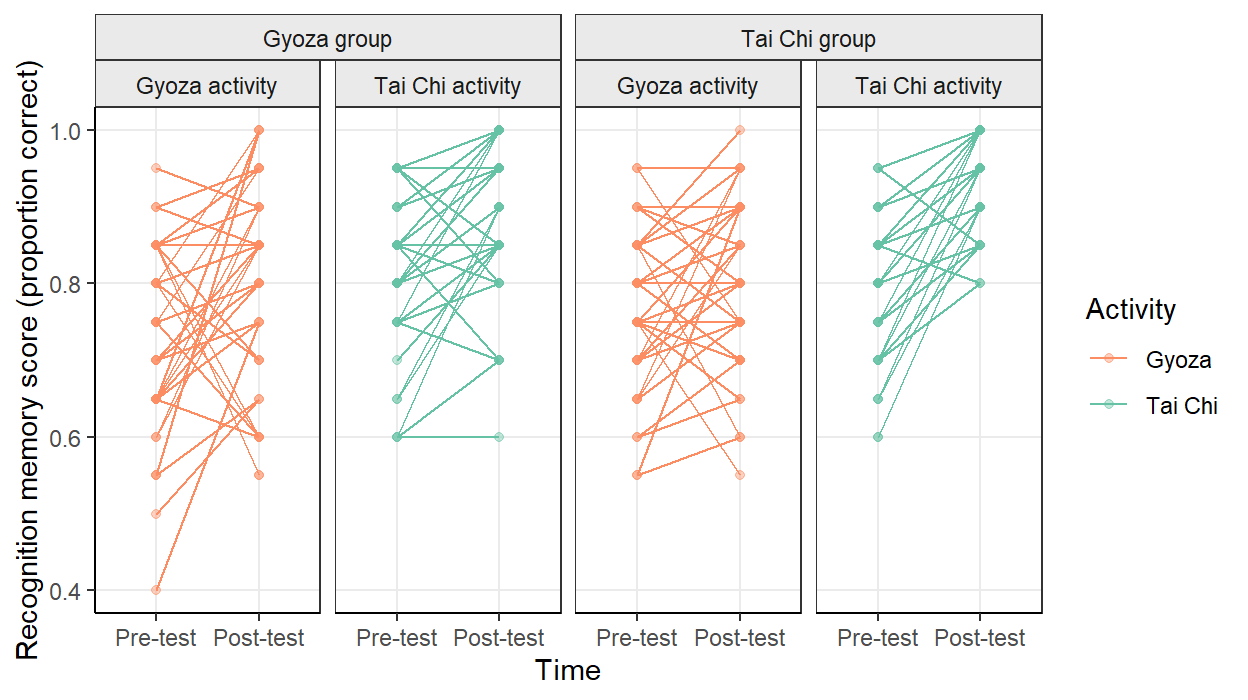


*Figure* S5. Raw data for recognition memory performance in terms of proportion correct. A: Dots represent individual data points; box charts and half-violin plots illustrate the spread of the raw data. B: Lines connect data from the same participant.

A.

**
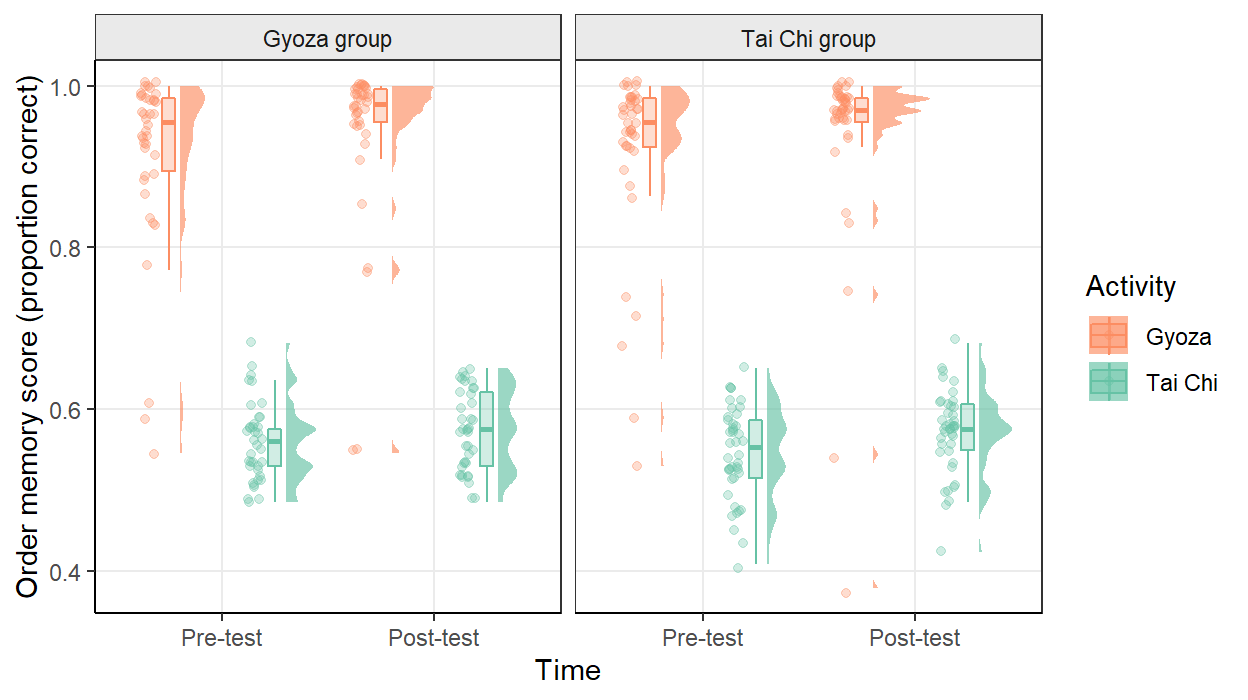
**

B.


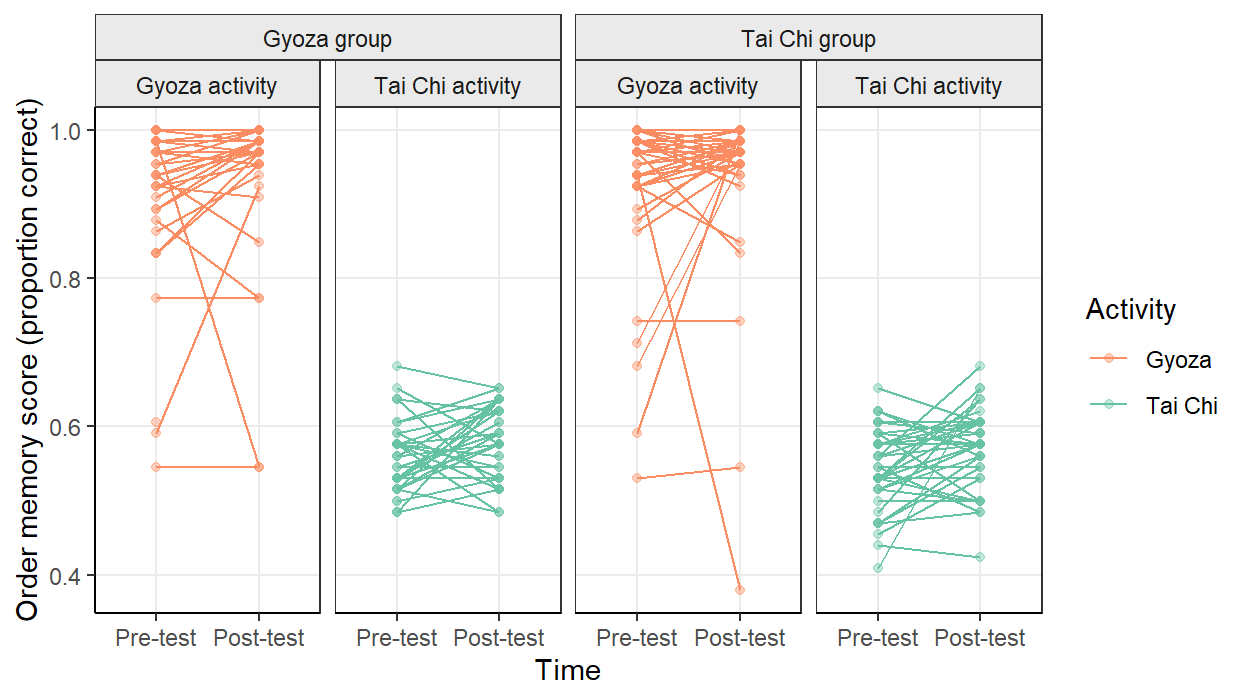


*Figure* S6. Raw data for order memory performance in terms of proportion correct. A: Dots represent individual data points; box charts and half-violin plots illustrate the spread of the raw data. B: Lines connect data from the same participant.


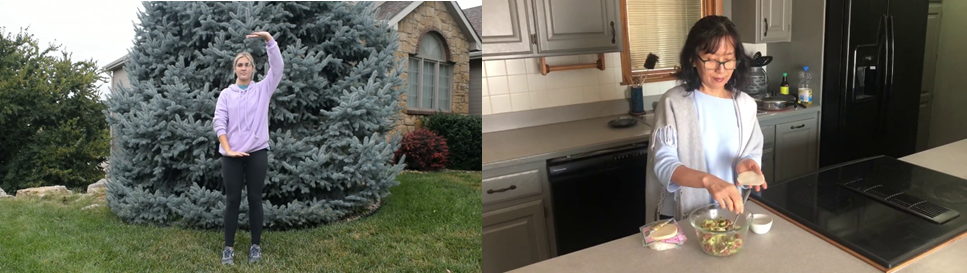


*Figure* S7. Screenshots from the pre- and post-test videos for each activity.


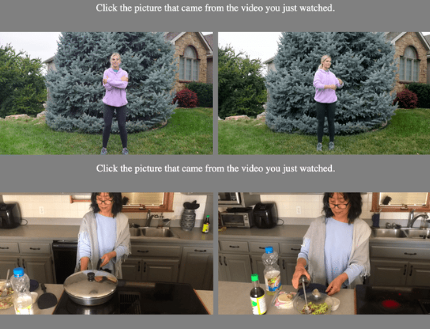

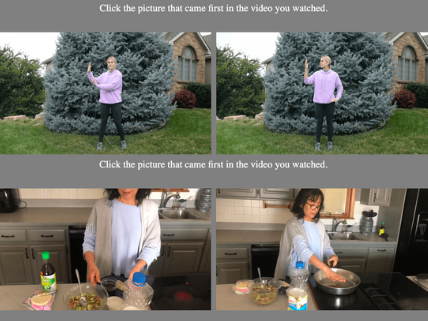
*Figure* S8. Screenshots from the recognition (left panel) and order memory task (right panel).


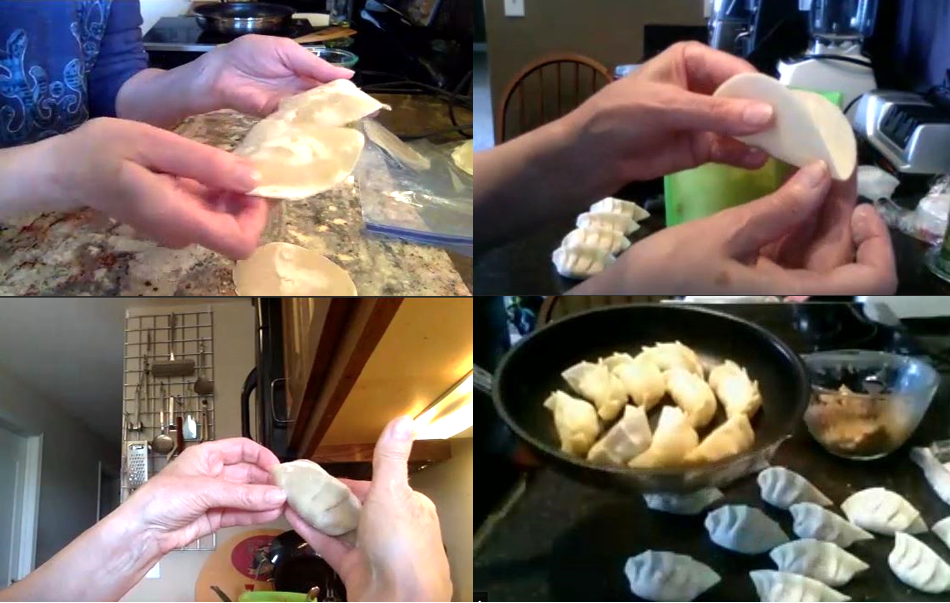


*Figure* S9. Screenshots from a live gyoza-making workshop.


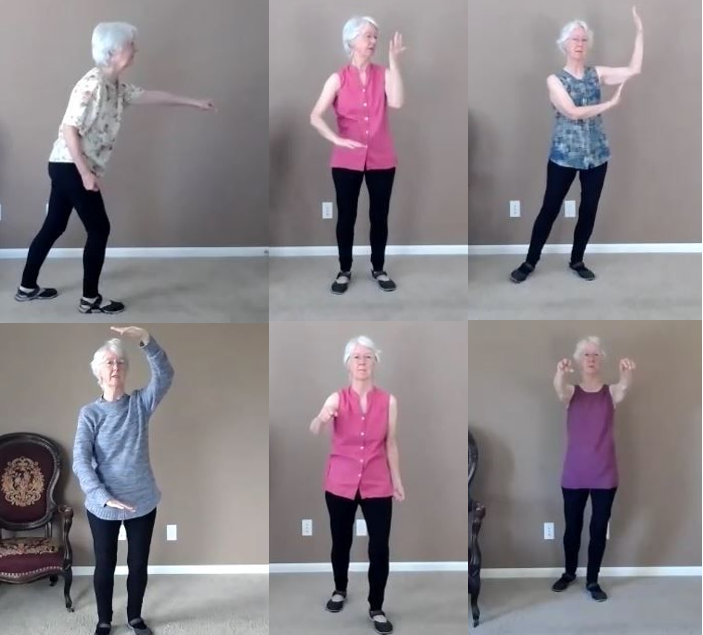


*Figure* S10. Screenshots from a live Tai chi workshop.

Table S1

*Fixed effects parameter estimates of the multilevel binomial logistic regression with Group, Time, Activity, and their interactions and random effects of Participant (intercept effect) and Time (slope effect) predicting the percentage correct on an expertise survey. All categorical variables are effect coded.*

|  | *B* | *SE* | *z*-value | *p*-value |  |
| --- | --- | --- | --- | --- | --- |
| (Intercept) | -0.76 | 0.08 | -9.43 | 0.00 | *** |
| Group[Gyoza] | -0.12 | 0.08 | -1.52 | 0.13 |  |
| Time[Pre-Test] | -1.14 | 0.05 | -24.18 | 0.00 | *** |
| Activity[Gyoza] | -0.35 | 0.04 | -9.61 | 0.00 | *** |
| Group[Gyoza]:Time[Pre-Test] | 0.03 | 0.05 | 0.57 | 0.57 |  |
| Group[Gyoza]:Activity[Gyoza] | 0.42 | 0.04 | 11.40 | 0.00 | *** |
| Time[Pre-Test]:Activity[Gyoza] | -0.16 | 0.04 | -4.46 | 0.00 | *** |
| Group[Gyoza]:Time[Pre-Test]:Activity[Gyoza] | -0.39 | 0.04 | -10.71 | 0.00 | *** |

Note: Signif. codes: 0 <= '***' < 0.001 < '**' < 0.01 < '*' < 0.05

Table S2

*Fixed effects parameter estimates of the multilevel Poisson regression with Group, Time, Activity, and their interactions, along with a random intercept effect of Participant, and a random slope effect of Time, predicting the number of times users segmented. All categorical variables are effect coded.*

|  | *B* | *SE* | *z*-value | *p*-value |  |
| --- | --- | --- | --- | --- | --- |
| (Intercept) | 2.86 | 0.07 | 42.04 | 0.00 | *** |
| Group[Gyoza] | 0.05 | 0.07 | 0.68 | 0.49 |  |
| Time[Pre-Test] | 0.07 | 0.03 | 1.97 | 0.05 | * |
| Activity[Gyoza] | -0.26 | 0.01 | -20.45 | 0.00 | *** |
| Group[Gyoza]:Time[Pre-Test] | -0.04 | 0.03 | -1.13 | 0.26 |  |
| Group[Gyoza]:Activity[Gyoza] | -0.09 | 0.01 | -7.11 | 0.00 | *** |
| Time[Pre-Test]:Activity[Gyoza] | -0.02 | 0.01 | -1.53 | 0.13 |  |
| Group[Gyoza]:Time[Pre-Test]:Activity[Gyoza] | -0.02 | 0.01 | -1.34 | 0.18 |  |

Note: Signif. codes: 0 <= '***' < 0.001 < '**' < 0.01 < '*' < 0.05

Table S3

*Fixed effects parameter estimates of the multilevel linear model with Group, Time, Activity, and their interactions, along with Segmentation Count and a random intercept effect of Participant, predicting segmentation agreement. All categorical variables are effect coded.*

|  | *B* | *SE* | *z*-value | *p*-value |  |
| --- | --- | --- | --- | --- | --- |
| (Intercept) | 0.36 | 0.01 | 26.85 | 0.00 | *** |
| Group[Gyoza] | 0.01 | 0.01 | 0.84 | 0.40 |  |
| Time[Pre-Test] | -0.01 | 0.01 | -1.76 | 0.08 | . |
| Activity[Gyoza] | -0.08 | 0.01 | -12.25 | 0.00 | *** |
| Segmentation Count | -0.00 | 0.00 | -10.03 | 0.00 | *** |
| Group[Gyoza]:Time[Pre-Test] | 0.01 | 0.01 | 0.82 | 0.41 |  |
| Group[Gyoza]:Activity[Gyoza] | -0.01 | 0.01 | -2.08 | 0.04 | * |
| Time[Pre-Test]:Activity[Gyoza] | 0.01 | 0.01 | 2.00 | 0.05 | * |
| Group[Gyoza]:Time[Pre-Test]:Activity[Gyoza] | -0.01 | 0.01 | -1.00 | 0.32 |  |

Note: Signif. codes: 0 <= '***' < 0.001 < '**' < 0.01 < '*' < 0.05

Table S4

*Fixed effects parameter estimates of the multilevel binomial logistic model with Group, Time, Activity, and their interactions and random effects of Participant (intercept effect) and Time (slope effect) predicting our recall composite variable. All categorical variables are effect coded.*

|  | *B* | *SE* | *z*-value | *p*-value |  |
| --- | --- | --- | --- | --- | --- |
| (Intercept) | -2.36 | 0.17 | -13.68 | 0.00 | *** |
| Group[Gyoza] | -0.07 | 0.17 | -0.40 | 0.69 |  |
| Time[Pre-Test] | 0.07 | 0.08 | 0.79 | 0.43 |  |
| Activity[Gyoza] | 0.50 | 0.02 | 22.64 | 0.00 | *** |
| Group[Gyoza]:Time[Pre-Test] | -0.12 | 0.08 | -1.52 | 0.13 |  |
| Group[Gyoza]:Activity[Gyoza] | 0.09 | 0.02 | 3.90 | 0.00 | *** |
| Time[Pre-Test]:Activity[Gyoza] | 0.12 | 0.02 | 5.63 | 0.00 | *** |
| Group[Gyoza]:Time[Pre-Test]:Activity[Gyoza] | -0.09 | 0.02 | -3.85 | 0.00 | *** |

Note: Signif. codes: 0 <= '***' < 0.001 < '**' < 0.01 < '*' < 0.05

Table S5

*Fixed effects parameter estimates of the multilevel binomial logistic model with Group, Time, Activity, and their interactions, with a random intercept effect of Participant and a random slope effect of Time, predicting recognition performance All categorical variables are effect coded.*

|  | *B* | *SE* | *z*-value | *p*-value |  |
| --- | --- | --- | --- | --- | --- |
| (Intercept) | 1.62 | 0.06 | 28.55 | 0.00 | *** |
| Group[Gyoza] | -0.09 | 0.06 | -1.68 | 0.09 | . |
| Time[Pre-Test] | -0.36 | 0.04 | -8.34 | 0.00 | *** |
| Activity[Gyoza] | -0.35 | 0.04 | -10.04 | 0.00 | *** |
| Group[Gyoza]:Time[Pre-Test] | 0.07 | 0.04 | 1.71 | 0.09 | . |
| Group[Gyoza]:Activity[Gyoza] | 0.05 | 0.04 | 1.52 | 0.13 |  |
| Time[Pre-Test]:Activity[Gyoza] | 0.18 | 0.04 | 5.15 | 0.00 | *** |
| Group[Gyoza]:Time[Pre-Test]:Activity[Gyoza] | -0.14 | 0.04 | -3.94 | 0.00 | *** |

Note: Signif. codes: 0 <= '***' < 0.001 < '**' < 0.01 < '*' < 0.05

Table S6

*Fixed effects parameter estimates of the binomial logistic model with Group, Time, Activity, and their interactions, with a random intercept effect of Participant and a random slope effect of Time, predicting order memory performance. All categorical variables are effect coded.*

|  | *B* | *SE* | *z*-value | *p*-value |  |
| --- | --- | --- | --- | --- | --- |
| (Intercept) | 1.44 | 0.04 | 35.60 | 0.00 | *** |
| Group[Gyoza] | 0.00 | 0.04 | 0.05 | 0.96 |  |
| Time[Pre-Test] | -0.11 | 0.03 | -3.95 | 0.00 | *** |
| Activity[Gyoza] | 1.18 | 0.02 | 54.10 | 0.00 | *** |
| Group[Gyoza]:Time[Pre-Test] | -0.01 | 0.03 | -0.34 | 0.74 |  |
| Group[Gyoza]:Activity[Gyoza] | -0.02 | 0.02 | -0.84 | 0.40 |  |
| Time[Pre-Test]:Activity[Gyoza] | -0.07 | 0.02 | -3.10 | 0.00 | ** |
| Group[Gyoza]:Time[Pre-Test]:Activity[Gyoza] | -0.02 | 0.02 | -0.83 | 0.41 |  |

Note: Signif. codes: 0 <= '***' < 0.001 < '**' < 0.01 < '*' < 0.05

Table S7

*Means, standard deviations, and zero-order correlations with confidence intervals*

| Variable | *M* | *SD* | 1 | 2 | 3 | 4 | 5 |
| --- | --- | --- | --- | --- | --- | --- | --- |
| 1. Recog | 0.81 | 0.12 |  |  |  |  |  |
| 2. Order | 0.74 | 0.20 | -.28** |  |  |  |  |
|  |  |  | [-.38, -.17] |  |  |  |  |
| 3. ACS A1 | 0.09 | 0.11 | .18** | .23** |  |  |  |
|  |  |  | [.06, .29] | [.12, .34] |  |  |  |
| 4. ACS A2 | 0.24 | 0.22 | .03 | .43** | .84** |  |  |
|  |  |  | [-.08, .15] | [.33, .52] | [.80, .87] |  |  |
| 5. Survey | 0.37 | 0.29 | .29** | -.11 | .06 | -.01 |  |
|  |  |  | [.18, .40] | [-.22, .01] | [-.06, .17] | [-.13, .11] |  |
| 6. SegAgree | 0.29 | 0.17 | .22** | -.30** | .02 | -.09 | .19** |
|  |  |  | [.10, .33] | [-.41, -.19] | [-.10, .13] | [-.20, .03] | [.07, .30] |

*Note.* *M* and *SD* are used to represent mean and standard deviation, respectively. Values in square brackets indicate the 95% confidence interval for each correlation. * indicates *p* < .05. ** indicates *p* < .01. Recog = recognition memory performance. Order = order memory performance. ACS A1 = number of A1 units in free recall response. ACS A2 = number of A2 units in free recall response. Survey = expertise survey performance. SegAgree = Segmentation agreement compared to the knowledgeable group in post-test.

Expert Screening Survey

The following questions assess your knowledge about cooking. For each question, mark your answer by clicking on the appropriate response. If you do not know the answer to a question, you may choose “I don’t know.” Please do **not** use outside sources to answer the questions.

Q1 Who does the cooking in your household?

- I do (3)
- Another member of the household (2)
- I take turns cooking (1)
- I do not eat at home (0)

Q2 What foods have you made in the past? (Select all that apply)

▢ Stuffed calamari (1)

▢ Indian curry (0)

▢ Sushi (0)

▢ Dumplings (1)

▢ Homemade stuffed pasta (1)

▢ Wontons (1)

Q3 Where do gyozas originate?

- China (1)
- South Korea (0)
- Japan (0)
- Vietnam (0)
- I don't know (0)

Q4 Where are gyozas most popular?

- The United States (0)
- Japan (1)
- Vietnam (0)
- India (0)
- I don't know (0)

Q5 T/F: All dumplings are made the same.

- True (0)
- False (1)

Q6 Around how much filling should you put in a gyoza?

- A teaspoon (0)
- A tablespoon (1)
- One fourth cup (0)
- One half cup (0)
- I don't know (0)

Q7 What shape should a gyoza be?

- Completely round (0)
- Like a cylinder (0)
- Like a half moon (1)
- There is no correct shape (0)
- I don't know (0)

Q8 What helps seal the gyoza dough edges after they are filled?

- Olive oil (0)
- Water (1)
- Soy sauce (0)
- You do not need anything to seal the edges (0)
- I don't know (0)

Q9 How many pleats should be on a gyoza?

- Two (0)
- Four (1)
- About five (0)
- Ten (0)
- I don't know (0)

Q10 What method of cooking is used in traditional gyoza making?

- Steaming (0)
- Frying (0)
- Stewing (0)
- A combination of frying and steaming (1)
- I don't know (0)

Q11 What is the most common filling for gyozas?

- Ground beef and cabbage (0)
- Ground turkey and chives (0)
- Only vegetables (0)
- Ground pork and cabbage (1)
- I don't know (0)

Q12 What is typically served with gyozas?

- Soy sauce (1)
- Soup broth (0)
- Curry (0)
- Rice (0)
- Nothing (0)
- I don't know (2)

Q13 What is the gyoza dough typically referred to as?

- Baozi (0)
- Dim Sum (0)
- A Wrapper (1)
- Wonton (0)
- I don't know (0)

Q14 About how long does it take to cook an average gyoza?

- Ten minutes (0)
- Fifteen minutes (0)
- Up to an hour (0)
- There is no set minimum time (1)
- I don't know (0)

Q15 What is the difference between a jiaozi and a gyoza?

- Method of cooking (1)
- Method of preparation (2)
- Gyozas are a much newer dish (3)
- All of the above (4)
- There is no difference (5)
- I don't know (7)

Q16 What would NOT be found in a gyoza?

- Ginger (0)
- Cornstarch (0)
- Pork (0)
- Rice (1)
- I don't know (0)

Q17 To what thickness should the gyoza dough be rolled?

- 1-2 mm (1)
- 3 mm (0)
- 5 mm (0)
- There is not a specific thickness of the dough (0)
- I don't know (0)

Q18 With what is the gyoza dough typically made?

- Rice flour (0)
- Cornstarch (0)
- Corn flour (0)
- Wheat flour (1)
- I don't know (0)

Q19 What is the typical shape the gyoza dough?

- Square (0)
- Diamond (0)
- Circular (1)
- Rectangular (0)
- I don't know (0)

Q20 How would you rate yourself in gyoza making?

- No experience at all (0)
- Some experience (1)
- Intermediately experienced (2)
- Very experienced (3)
- Expert level experience (4)

The following questions assess your knowledge about Tai Chi. For each question, mark your answer by clicking on the appropriate response. If you do not know the answer to a question, you may choose “I don’t know.” Please do not use outside sources to answer the questions.

Q21 How experienced are you with any form of martial arts?

- No experience at all (0)
- Some experience (1)
- Intermediate experience (2)
- Very experienced (3)
- Expert level experience (4)

Q22 How experienced are you with any form of Yoga?

- No experience at all (0)
- Some experience (1)
- Intermediate experience (2)
- Very experienced (3)
- Expert level experience (4)

Q23 Which of these are Tai Chi styles?

- Yang-Style, Sun Style, Chen-Style (1)
- Wu-Style, Yang-Style, Wan-Style (0)
- Hao-Style, Chung-Style, Yume-Style (0)
- All of the above (0)
- I don't know (0)

Q24 What is/are the core tenet(s) of Tai Chi?

- Mindfulness (0)
- Circular movements (0)
- Controlled breathing (0)
- All of the above (1)
- I don't know (0)

Q25 What is/ are benefit(s) of Tai Chi

- Strengthen muscles (0)
- Encourages circular movement (0)
- Improves balance (0)
- All of the above (1)
- None of the above (0)
- I don't know (0)

Q26 In what country does Tai Chi originate?

- Japan (0)
- Taiwan (0)
- China (1)
- India (0)
- I don't know (0)

Q27 How often should you do Tai Chi?

- Daily (1)
- Weekly (0)
- Monthly (0)
- Bi-Monthly (0)
- I don't know (0)

Q28 T/F: As you improve your Tai Chi, your movements become much faster.

- True (1)
- False (0)

Q29 What kind of activity is Tai Chi?

- A team sport (0)
- A type of Yoga (0)
- A competitive sport (0)
- A Martial Art (1)
- I don't know (0)

Q30 T/F: You must be able to stand to practice Tai Chi

- True (0)
- False (1)

Q31 In more recent years, Tai Chi has become an exercise to:

- Promote self defense (0)
- Spread the knowledge of historical practices (0)
- Encourage low-stress exercise (1)
- Be practiced as a competitive sport (0)
- I don't know (0)

Q32 When did Tai Chi begin being practiced and taught in the United States?

- 1975 (0)
- The early 1900s (0)
- 1939 (1)
- It has always been practiced and taught in the United States (0)
- I don't know (2)

Q33 How many major styles of Tai Chi exist?

- One (0)
- Three (1)
- Five (0)
- Ten (0)
- I don't know (0)

Q34 Which aspect(s) guide the practice of Tai Chi?

- Health (0)
- Meditation (0)
- Martial Art (0)
- All of the above (1)
- None of the above (0)
- I don't know (2)

Q35 What symbol typically represents Tai Chi?

- The Sun (0)
- The Moon (0)
- The Yin and Yang (1)
- All of the above (0)
- I don't know (0)

Q36 When practicing Tai Chi, what should be emphasized?

- Rapid movement (0)
- Burning calories (0)
- Mindfulness (1)
- Competition (0)
- I don't know (0)

Q37 In what order should one warm up the body for practice?

- From toe to head (0)
- From head to toe (1)
- The order is not significant (0)
- You do not need to warm up for Tai Chi (0)
- I don't know (0)

Q38 T/F: Tai Chi is a practice that can be fully mastered.

- True (0)
- False (1)

Q39 How experienced are you in Tai Chi?

- Not at all experienced (0)
- Beginner level experience (1)
- Intermediate level experience (2)
- Very experienced (3)
- Expert level experience (4)
